# Prevalence and prognostic associations of cardiac abnormalities among hospitalized patients with COVID-19: a systematic review and meta-analysis

**DOI:** 10.1038/s41598-021-87961-x

**Published:** 2021-04-19

**Authors:** Louie F. Dy, Ryan C. V. Lintao, Cynthia P. Cordero, Ian Theodore G. Cabaluna, Leonila F. Dans

**Affiliations:** 1 College of Medicine, University of the Philippines Manila, 1000 Manila, Philippines; 2 Department of Clinical Epidemiology, College of Medicine, University of the Philippines Manila, 1000 Manila, Philippines; 3 Asia Pacific Center for Evidence-Based Healthcare, 1000 Manila, Philippines; 4 University of the Philippines COVID-19 Pandemic Response Team, University of the Philippines Resilience Institute, Manila, Philippines

**Keywords:** Epidemiology, Outcomes research

## Abstract

Although most patients recover from COVID-19, it has been linked to cardiac, pulmonary, and neurologic complications. Despite not having formal criteria for its diagnosis, COVID-19 associated cardiomyopathy has been observed in several studies through biomarkers and imaging. This study aims to estimate the proportion of COVID-19 patients with cardiac abnormalities and to determine the association between the cardiac abnormalities in COVID-19 patients and disease severity and mortality. Observational studies published from December 1, 2019 to September 30, 2020 were obtained from electronic databases (PubMed, Embase, Cochrane Library, CNKI) and preprint servers (medRxiv, bioRxiv, ChinaXiv). Studies that have data on prevalence were included in the calculation of the pooled prevalence, while studies with comparison group were included in the calculation of the odds ratio. If multiple tests were done in the same study yielding different prevalence values, the largest one was used as the measure of prevalence of that particular study. Metafor using R software package version 4.0.2 was used for the meta-analysis. A total of 400 records were retrieved from database search, with 24 articles included in the final analysis. Pooled prevalence of cardiac abnormalities in 20 studies was calculated to be 0.31 [95% Confidence Intervals (CI) of (0.23; 0.41)], with statistically significant heterogeneity (percentage of variation or I-squared statistic I^2^ = 97%, p < 0.01). Pooled analysis of 19 studies showed an overall odds ratio (OR) of 6.87 [95%-CI (3.92; 12.05)] for cardiac abnormalities associated with disease severity and mortality, with statistically significant heterogeneity (I^2^ = 85%, between-study variance or tau-squared statistic τ^2^ = 1.1485, p < 0.01). Due to the high uncertainty in the pooled prevalence of cardiac abnormalities and the unquantifiable magnitude of risk (although an increased risk is certain) for severity or mortality among COVID-19 patients, much more long-term prognostic studies are needed to check for the long-term complications of COVID-19 and formalize definitive criteria of “COVID-19 associated cardiomyopathy”.

## Introduction

Coronavirus Disease 2019 (COVID-19), caused by the novel virus Severe Acute Respiratory Syndrome Coronavirus 2 (SARS-CoV-2), has evidently spread throughout the world, claiming at least 1.1 million lives as of this writing^1^. While much of information regarding this pathogen, such as its transmission dynamics, spectrum of clinical manifestations, complications, diagnostics, and treatment have been determined.

Meanwhile, short-term and long-term effects of COVID-19 are still being elucidated. Although majority of the COVID-19 cases are mild and asymptomatic, and most patients recover from the disease, COVID-19 has been linked to cardiac, pulmonary and neurologic complications. As of this writing, there are reports of cardiac abnormalities and dysfunction, detected through biomarkers and imaging, among mild, moderate, severe, critical, and even recovered cases.

Cardiac pathology caused by SARS-CoV-2 has been documented in vitro^2^, and it was observed to be associated with worse outcomes. However, due to the variability in the overabundance of various prognostic studies, the definite proportion or frequency of this occurring among patients, and the definite magnitude of risk for severity and mortality have yet to be fully elucidated. There is also no definite criteria or formal definition of COVID-19 associated cardiomyopathy.

This study aims to estimate the proportion of COVID-19 patients with cardiac abnormalities and to determine the association between the cardiac abnormalities in COVID-19 patients and disease severity and mortality.

## Methodology

### Research question formulation, inclusion and exclusion criteria

The population of interest includes patients with laboratory-confirmed COVID-19 patients. The identified exposure is any form of cardiac abnormality found in diagnostic tests such as echocardiography, cardiac magnetic resonance imaging, electrocardiogram, and serum biomarkers such as troponin. These were compared to confirmed COVID-19 patients who have no abnormalities in aforementioned cardiac tests. The main outcomes of interest were severity, in-hospital mortality, or both. Both prospective and retrospective observational studies (cohort studies, case–control studies, case series) and randomized controlled studies were included, while all other study designs, such as case reports, commentaries, editorials, guidelines, reviews, and studies published only as abstracts were excluded.

### Literature search strategy

Comprehensive searches of electronic databases (PubMed, Embase, Cochrane Library, CNKI) and preprint servers (medRxiv, bioRxiv, ChinaXiv) were conducted, including studies published from December 1, 2019 to September 30, 2020 in both English and non-English languages. In the case of PubMed, search words include *("2019 nCoV" OR "2019nCoV" OR "2019-nCoV" OR "COVID 19" OR "COVID19" OR "COVID-19" OR "new coronavirus" OR "novel coronavirus" OR (Wuhan AND coronavirus) OR (Wuhan AND pneumonia) OR "SARS-CoV" OR "SARS-CoV-2" OR "SARS CoV-2")*, *("troponin" OR "cardiac" OR "myocardial" OR "TnI" OR "TnT" OR "cardiovascular" OR "heart" OR "CMR" OR "cardiac MRI" OR "cardiac magnetic resonance imaging" OR "echocardiography" OR "BNP" OR "brain natiuretic peptide" OR "NT-proBNP")*. In the Cochrane Library, search words include *("COVID-19" OR "coronavirus" OR "2019-nCoV") AND ("cardiac" OR "cardiovascular" OR "myocardial" OR "troponin" OR "echocardiography" OR "BNP" OR "NT-proBNP" OR "cardiac MRI" OR "CMR")*. In Chinese electronic databases, keywords include 新型冠状病毒, 心机损伤. *Free text search in Google Scholar used the queries "COVID-19 cardiac echocardiography", "COVID-19 cardiac troponin", "COVID-19 cardiac MRI".* If one search strategy, such as in the case of ChinaXiv, yielded no results, another approach was done to ensure no articles are missed out. Detailed step-by-step search strategy is elucidated in Supplementary Material 1.

### Study selection

**Figure 1:**
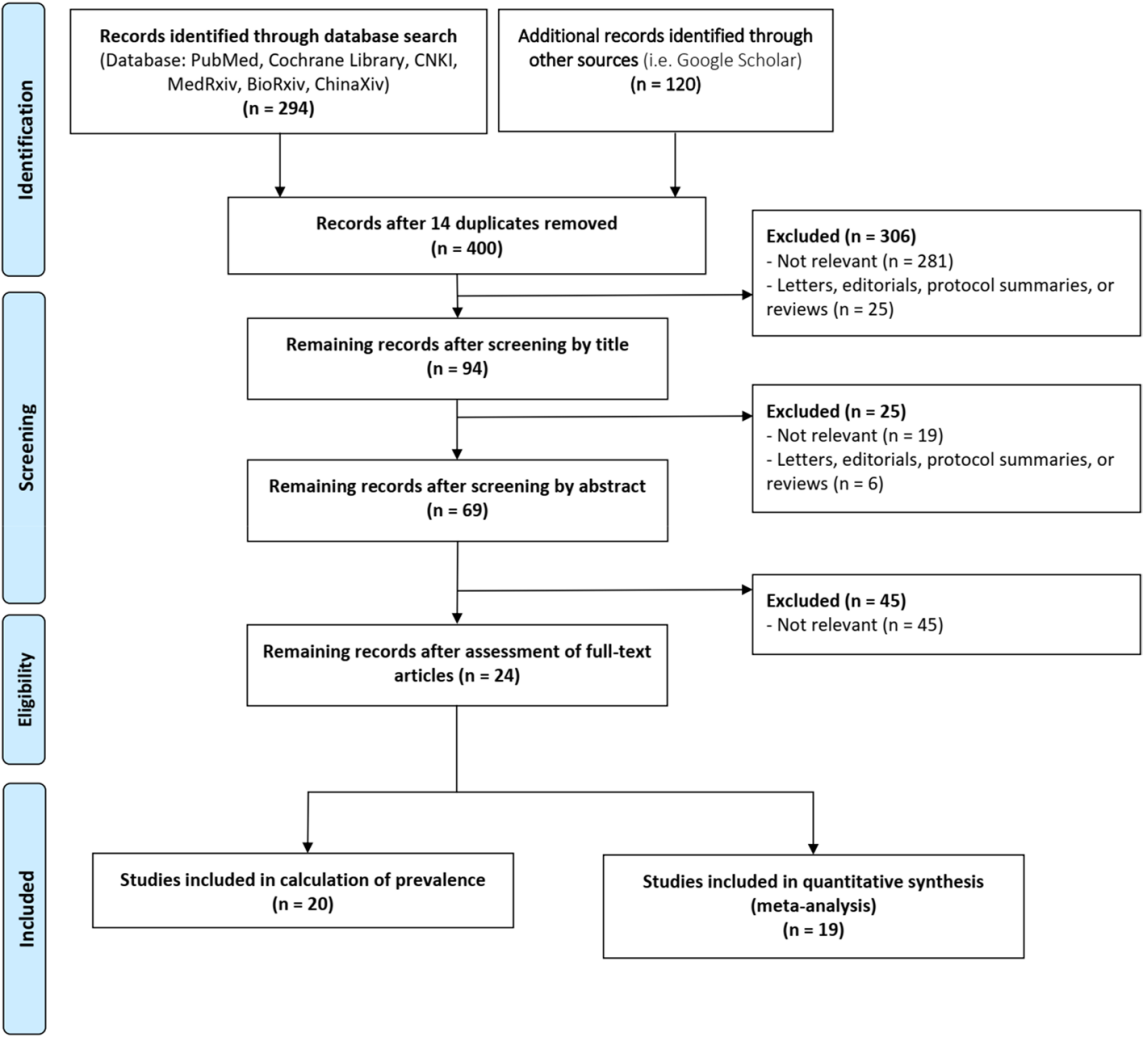
Preferred Reporting Items for Systematic Reviews and Meta-Analyses (PRISMA) flow diagram of this study, showing the studies included in pooled prevalence calculation and quantitative synthesis.

One assessor reviewed all relevant titles and abstracts independently and selected articles for full-text review if inclusion criteria are met. Full-text review and appraisal was done by two assessors. Disagreements were resolved by discussion between the two reviewers; a third person was involved when consensus cannot be reached (Fig. 1).

### Data extraction

The following data from each study were extracted: first author’s name, study design, population type (whether only severe and critical cases are included, or even mild and moderate cases are included), diagnostic methods for cardiac abnormalities, frequency of cardiac abnormalities, and frequencies of severity and mortality among patients with and without the aforementioned cardiac abnormalities. Studies that have data on prevalence were included in the calculation of the pooled prevalence, while studies with comparison group were included in the calculation of the odds ratio. If multiple tests were done in the same study yielding different prevalence values, the largest one was used as the measure of prevalence of that particular study.

### Risk of bias (quality) assessment for prognosis studies

Two reviewers independently assessed the quality of the included studies using the framework by Dans et al.^3^ that uses the following signaling questions answerable by a “Yes” or “No”:

- Were all important prognostic factors considered?
- Were unbiased criteria used to detect the outcome in all patients?
- Was follow-up rate adequate?
- If clinical prediction rules are being tested, was a separate validation study done?

Results from these ratings and especially non-agreement were then the basis for discussion until final consensus is made.

### Strategy for data synthesis

Meta-analysis software, Metafor (R package) by Viechtbauer^4^ using R software package version 4.0.2, was used in this study. Heterogeneity among the studies was assessed using the Cochran's Q and I^2^ statistics. Cochran’s Q is the result of the Chi-squared test of several studies^5^. At 0.10 significance level, p-values < 0.10 indicate rejection of the hypothesis that the measures of association are similar^5^. The I^2^ statistic represents the percentage of variability in effect estimates due to real dispersion among the studies^5^. I^2^ of at least 50% is considered substantial heterogeneity; it means that at least half of the total variability among effect sizes is due to true heterogeneity between studies^5^. The tau-squared statistic is a function of I^2^.

Random effects models^6^ were used to account for the heterogeneity of included studies. In studies with zero count for events in either the exposure or comparator group, 0.5 was automatically added in all counts^6^. Pooled prevalence of cardiac abnormalities was estimated at 95% confidence level using the Logit Transformation Method and Clopper–Pearson Intervals respectively. The Logit Transformation Method was used to estimate the pooled prevalence by log-transforming the prevalences of the individual studies^7^. The Clopper–Pearson Intervals, or more commonly known as the exact binomial test, calculates the confidence intervals based on the binomial distribution and therefore produces more conservative estimates and wide confidence intervals^8, 9^.

Pooled odds ratios and 95%-CI were calculated using the DerSimonian–Laird Method. The DerSimonian–Laird Method adjusts the standard errors of the individual odds ratios to incorporate variations across different studies^10^, producing wider confidence intervals.

A funnel plot, together with Egger’s Test, was used to determine potential publication bias. The results—the intercept, its confidence intervals—represent the degree of asymmetry of the funnel plot^11^. The farther it is from zero, the more asymmetric is the funnel, indicating publication bias^11^.

Post-hoc sensitivity analyses—leave-one-out analysis and Baujat diagnostics—were also done.

### Analysis of subgroups or subsets

Subgroup analyses were done according to the following.

1. Study design: case–control, cross-sectional, and cohort studies;
2. Study population: studies that included only severe and critical patients, and studies that included mild, moderate, severe, and critical patients as defined by the World Health Organization Interim Guidelines for the Clinical Management of COVID-19, or Novel Coronavirus Infectious Pneumonia Management Guidelines by the People’s Republic of China Central Health Committee; and
3. Type of cardiac test done.

## Results

### Study selection

From the database search, 294 articles were retrieved and additional 120 studies were identified through Google Scholar, giving a total of 400 studies after 14 duplicates were removed. After screening articles by title, 306 articles were excluded because 281 articles are not relevant or did not satisfy the inclusion criteria, and 25 articles were letters, editorials, protocol summaries, or reviews, leaving only 94 articles. After screening articles by abstract, 25 articles were excluded because 19 articles were not relevant, and 6 articles were letters, editorials, protocol summaries, or reviews. 69 articles then underwent full-text screening, thus excluding 45 articles, all of which do not have the relevant population, exposure, or outcome parameters. This leaves 24 articles to be included in the final analysis. 20 articles are included in estimation of the pooled prevalence, and 19 articles are included in the meta-analysis of odds ratios.

### Summary of characteristics of included studies

Among 24 papers included in the final analysis, there are four case–control studies, two cross-sectional studies, and 18 cohort studies. All studies were done in a hospitalized setting. Deng et al. had analysis on both severity and mortality. Most of these studies involved multiple cardiac biomarkers. More details of the included studies are found in Table 1.

**Table 1:** General characteristics of included studies.

Study	Month, Year	Study design	Sample size	Baseline severity	Age, in mean (SD) or median (IQR)	Males (%)	Tests involved
Cao et al.	July, 2020	Retrospective cohort	244	Moderate, severe, critical	62.58 (13.43)	133 (54.5%)	hsTnI
Chen et al.	March, 2020	Cross-sectional	150	Non-severe, severe	59 (16)	84 (56%)	cTnI, NT-proBNP
Cummings et al.	June, 2020	Prospective cohort	257	Critical	62 (51–72)	171 (67%)	hsTnT
Deng et al.	July, 2020	Retrospective cohort	112	Non-severe, severe	65.0 (49.0–70.8)	57 (50.9%)	cTnI > 0.04, cTnI > 0.12, ECG (ST elevation, ST-T wave changes), echocardiography (LVEF < 50%, TAPSE < 16 mm, pulmonary hypertension signs, pericardial effusion > 5 mm)
Gao et al.	April, 2020	Retrospective cohort	54	Severe	60.4 (16.1)	24 (44.4%)	NT-proBNP
Ge et al.	January, 2020	Prospective cohort	51	Severe, critical	70 (58.0–79.0)	37 (72.5%)	Echo (at least one abnormality)
Gil-Rodrigo et al.	August, 2020	Prospective cohort	1000	Not indicated	62 (18)	562 (56.2%)	cTnI, NT-proBNP
He et al.	June, 2020	Retrospective cohort	54	Severe, critical	68.0 (59.8–74.3)	34 (63%)	NT-proBNP
Hong et al.	March, 2020	Retrospective cohort	18	Severe, critical	63.5 (51.5–67.5)	9 (50%)	cTnI, CK, CK-MB, NT-proBNP
Huang et al.	August, 2020	Retrospective cohort	60	Severe	57 (26–97)	35 (58.3%)	cTnT, CK, CK-MB
Knight et al.	September, 2020	Case–control	828	Not indicated	NA	NA	hsTnT, CMR
Li et al.	August, 2020	Retrospective cohort	157	Not indicated	NA	NA	echocardiography (RV dysfunction, heart failure)
Liu et al.	January, 2020	Retrospective cohort	34	Not indicated	60.5 (40–80)	18 (52.9%)	BNP, cTnI
McCullough et al.	July, 2020	Retrospective cohort	756	Not indicated	64.0 (51.9–74.6)	478 (63.2%)	ECG (atrial fibrillation, PACs, PVCs, AV block first degree, AV block third degree, abnormal axis, RBBB, LBBB, nonspecific intraventricular block, LV hypertrophy, RV hypertrophy, MI age undetermined, ST elevation, T-wave inversion, nonspecific repolarization abnormality)
Nie et al.	September, 2020	Case–control	311	Not indicated	63 (54–70)	190 (61.1%)	cTnI
Pagnesi et al.	September, 2020	Cross-sectional	200	Not indicated	62 (55–74)	131 (65.5%)	echocardiography (RV dysfunction, pulmonary hypertension signs)
Rath et al.	June, 2020	Prospective cohort	123	Not indicated	68 (15)	77 (62.6%)	cTnI, NT-proBNP, echocardiography (LV hypertrophy, visually estimated impaired RV function, TAPSE < 20 mm, aortic stenosis, aortic regurgitation, mitral regurgitation, tricuspid regurgitation, pericardial effusion), ECG (RBBB, LBBB, negative T-wave, ST depression)
Shi et al.	June, 2020	Retrospective cohort	671	Severe	63 (50–72)	322 (48.0%)	cTnI, CK-MB, myoglobin
Szekely et al.	July, 2020	Prospective cohort	100	Mild, moderate, severe	66.1 (17.3)	63 (63%)	echocardiography (combined, RV volume overload, pulmonary acceleration time), ECG (long QT, T-wave inversion, ST segment depression, ST segment elevation, LBBB, RBBB, atrial fibrillation), cTnI, BNP
Li et al.	June, 2020	Case–control	227	Mild, moderate, severe, critical	55 (17)	129 (56.8%)	myoglobin, cTn, CK-MB, BNP
Xu et al.	September, 2020	Case–control	102	Not indicated	NA	NA	TNT-HSST
Zhang et al.	May, 2020	Retrospective cohort	30	ICU, non-ICU	56.0 (42.0–68.0)	67 (49.6%)	cTnT
Zhou et al.	March, 2020	Retrospective cohort	145	General, severe, critical	56.0 (46.0–67.0)	119 (62%)	hsTnI, CK
Zou et al.	August, 2020	Retrospective cohort	154	Mostly ICU	60.68 (13.00)	67 (43.51%)	hsTnI, CK

### Summary of appraisal of included papers

Two (2) studies—Knight et al. and Li et al.—have unclear risk of bias because it is unclear whether they have considered all important prognostic factors (D1). The rest have low risk of bias overall. The risk of bias assessment traffic light plot can be seen in Supplementary Material 2.

The major weaknesses of these studies lie in their retrospective design, questionable temporality (as some are cross-sectional and case–control), different and sometimes unclear thresholds to define an “abnormal cardiac test”.

### Pooled prevalence calculation

**Figure 2:**
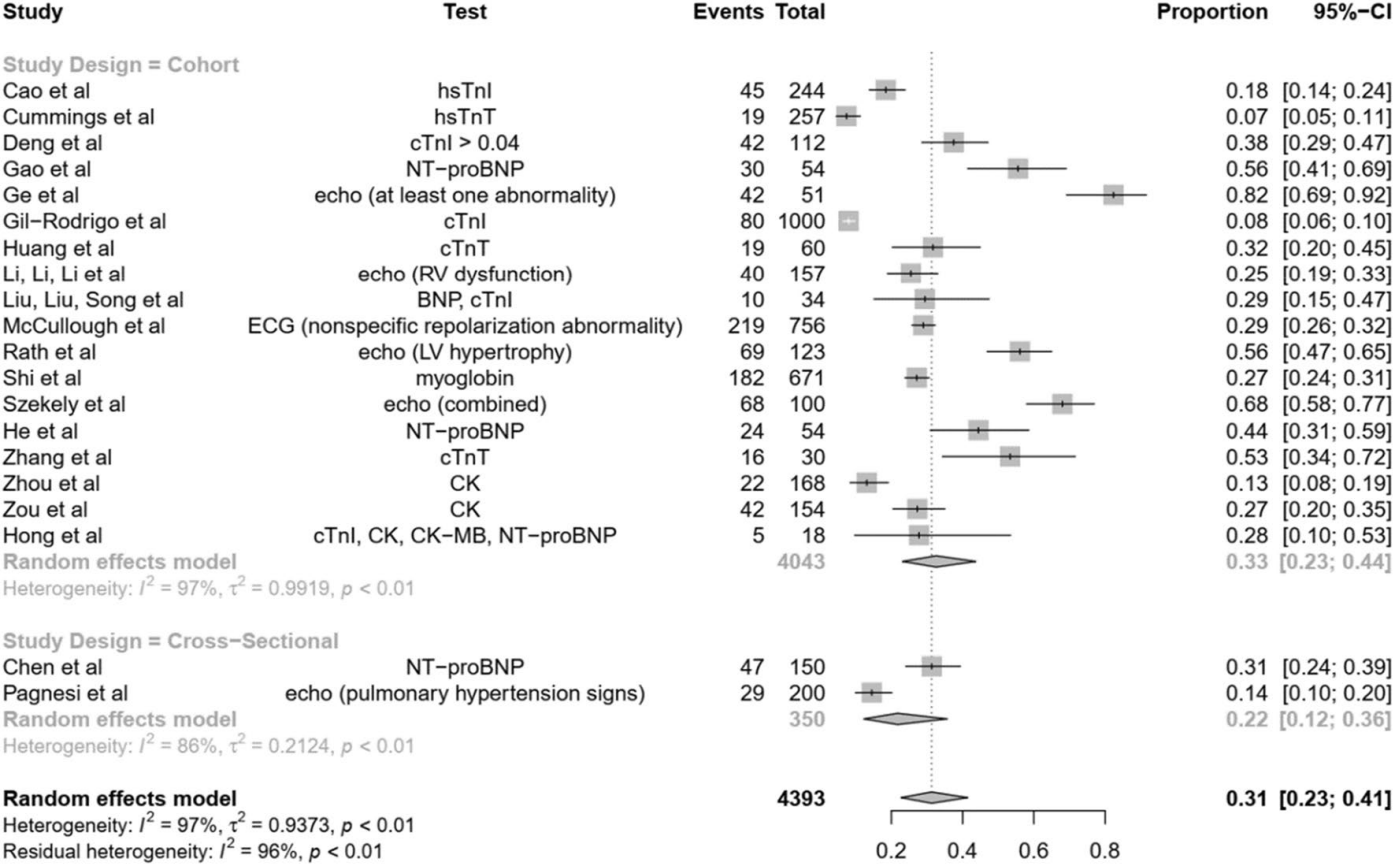
Forest plot showing the pooled prevalence of cardiac abnormalities in patients with COVID-19.

A total of 20 studies (two cross-sectional studies and 18 cohort studies) were included in the calculation of pooled prevalence, yielding a total of 4393 patients, 1040 of whom had at least one abnormal result in a cardiac test. Pooled prevalence is at 0.31 [95%-CI (0.23; 0.41)] (Fig. 2). Heterogeneity was statistically significant with I^2^ = 97%, τ^2^ = 0.9373, p < 0.01.

### Subgroup analysis according to study design

Breaking this down further according to study design, cohort studies report a pooled prevalence of 0.33 [95%-CI (0.23; 0.44)], and cross-sectional studies report a pooled prevalence of 0.22 [95%-CI (0.12, 0.36)]. Likewise, heterogeneity is significant for both subgroups, with I^2^ = 97%, τ^2^ = 0.9919, p < 0.01 in cohort studies and I^2^ = 86%, τ^2^ = 0.2124, p < 0.01 (Fig. 2).

### Association of cardiac abnormalities with disease severity and mortality

**Figure 3:**
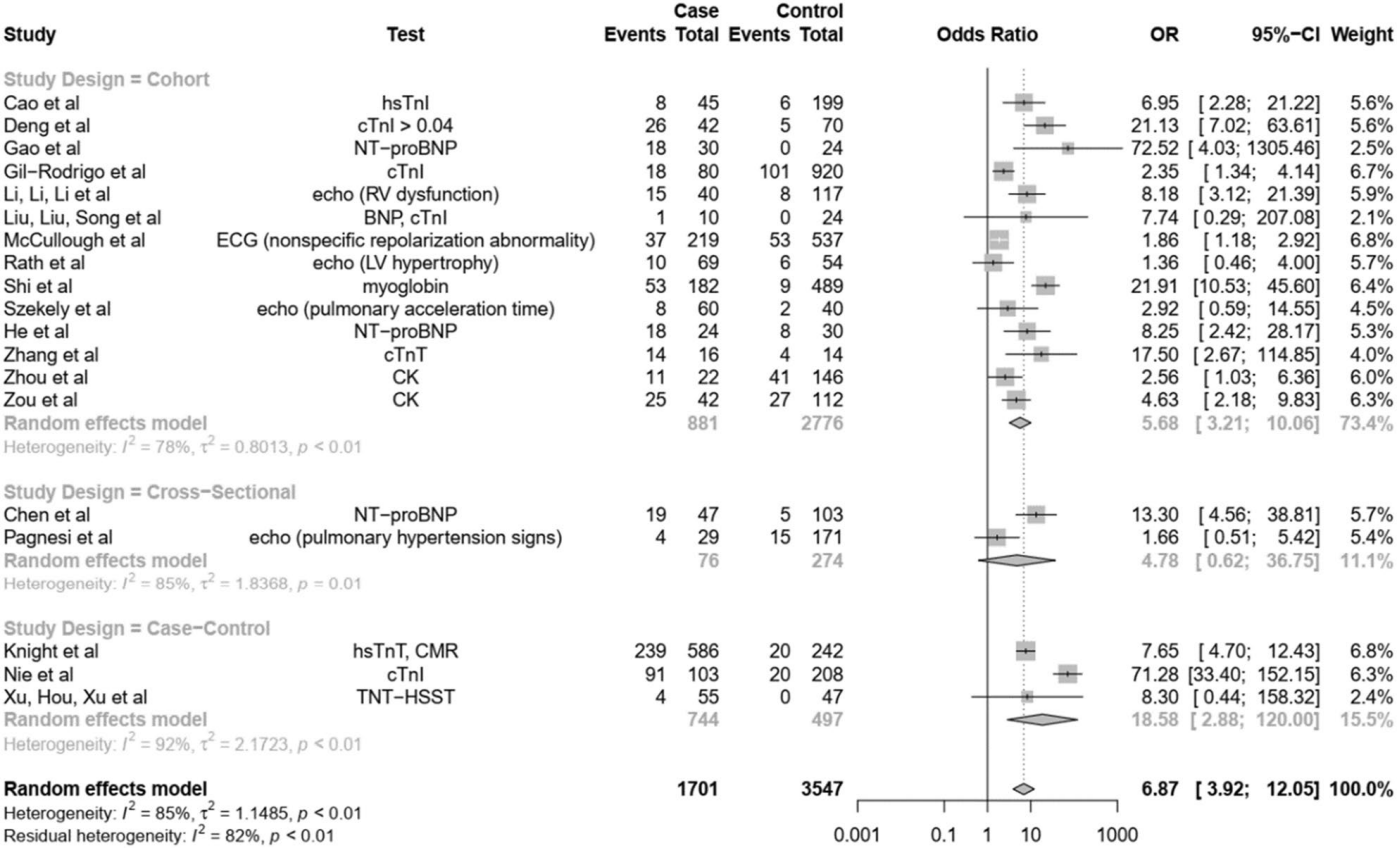
Forest plot of all 19 studies showing the odds ratio (OR) as well as subgroup analyses based on study design.

A total of 19 studies (two cross-sectional, three case–control, and 14 cohort studies) were included in the calculation of odds ratios. Pooled analysis of 19 studies showed an overall odds ratio (OR) of 6.87 [95%-CI (3.92; 12.05)] (Fig. 3) with significant heterogeneity (I^2^ = 85%, τ^2^ = 1.1485, p < 0.01).

### Subgroup analysis according to study design

Further subgroup analysis according to study design showed: (1) for cohort studies, an OR of 5.68 [95%-CI (3.21; 10.06)] with significant heterogeneity (I^2^ = 78, τ^2^ = 0.8013, p < 0.01); (2) for cross-sectional studies, an OR of 4.78 [95%-CI (0.62; 36.75)] with significant heterogeneity (I^2^ = 85, τ^2^ = 1.8368, p < 0.01); and (3) for case control studies, an OR of 18.58 [95%-CI (2.88; 120.00)] with significant heterogeneity (I^2^ = 92%, τ^2^ = 2.1723, p < 0.01).

### Subgroup analysis according to the type of test

**Figure 4:**
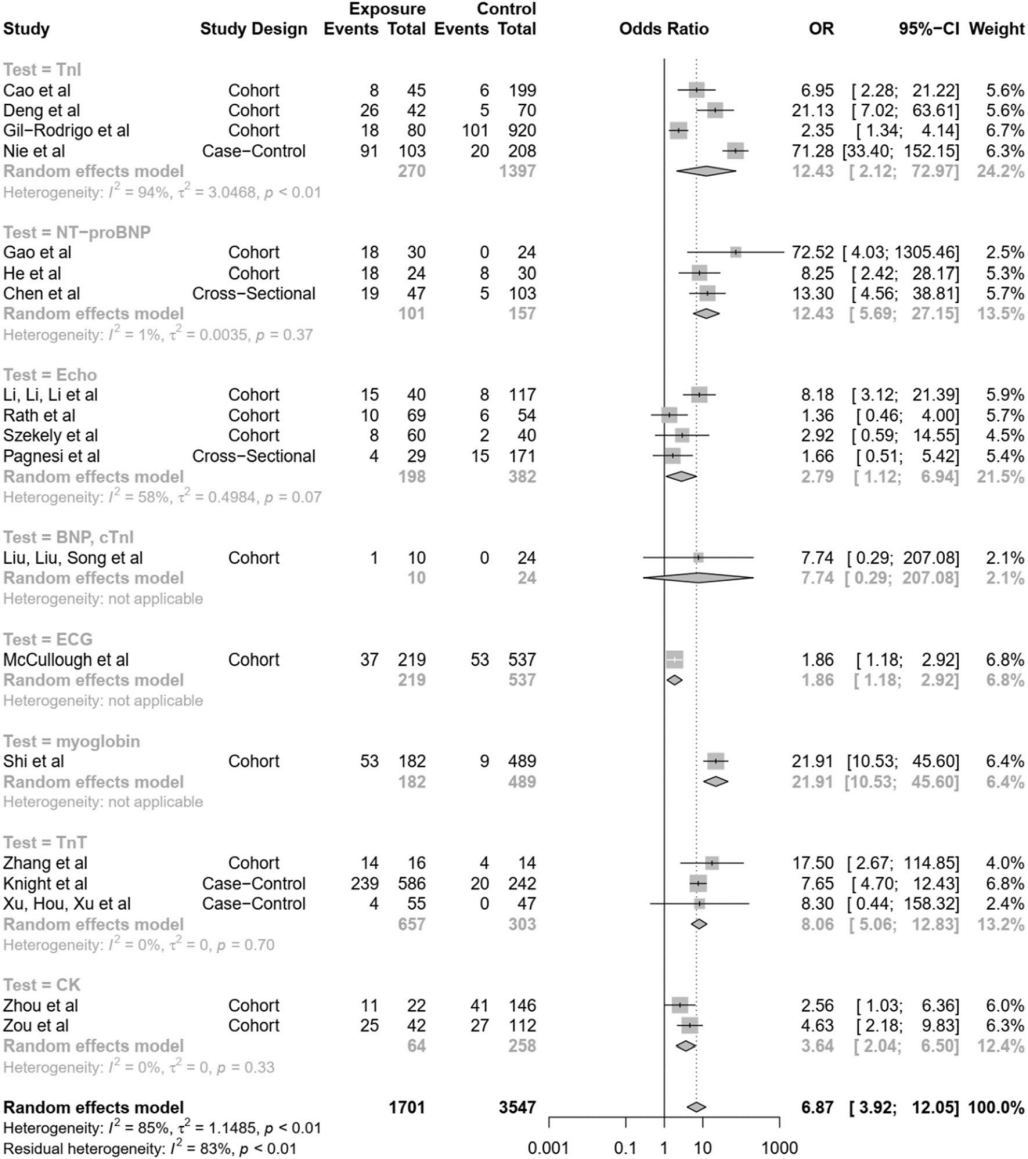
Forest plot of subgroup analyses according to type of test done.

Further subgroup analysis according to the type of test showed: for Troponin I (TnI), an OR of 12.43 [95%-CI (2.44; 19.77)] with significant heterogeneity (I^2^ = 94%, τ^2^ = 3.0468, p < 0.01); for NT-proBNP, an OR of 12.43 [95%-CI (5.69; 27.15)] with minimal heterogeneity (I^2^ = 1%, τ^2^ = 0.0035, p = 0.37); for 2D Echocardiography, an OR of 2.79 [95%-CI (1.12; 6.94)] with significant heterogeneity (I^2^ = 58%, τ^2^ = 0.4984, p = 0.07); for Troponin T (TnT), an OR of 8.06 [95%-CI (5.06; 12.83)] with minimal heterogeneity (I^2^ = 0%, τ^2^ = 0, p = 0.70); and for Creatinine Kinase (CK), an OR of 3.64 [95%-CI (2.04; 6.50)] with minimal heterogeneity (I^2^ = 0%, τ^2^ = 0, p = 0.33) (Fig. 4). Only one study was noted for BNP, ECG, and myoglobin; hence, the pooled OR and the heterogeneity could not be obtained.

### Publication bias of included studies

**Figure 5:**
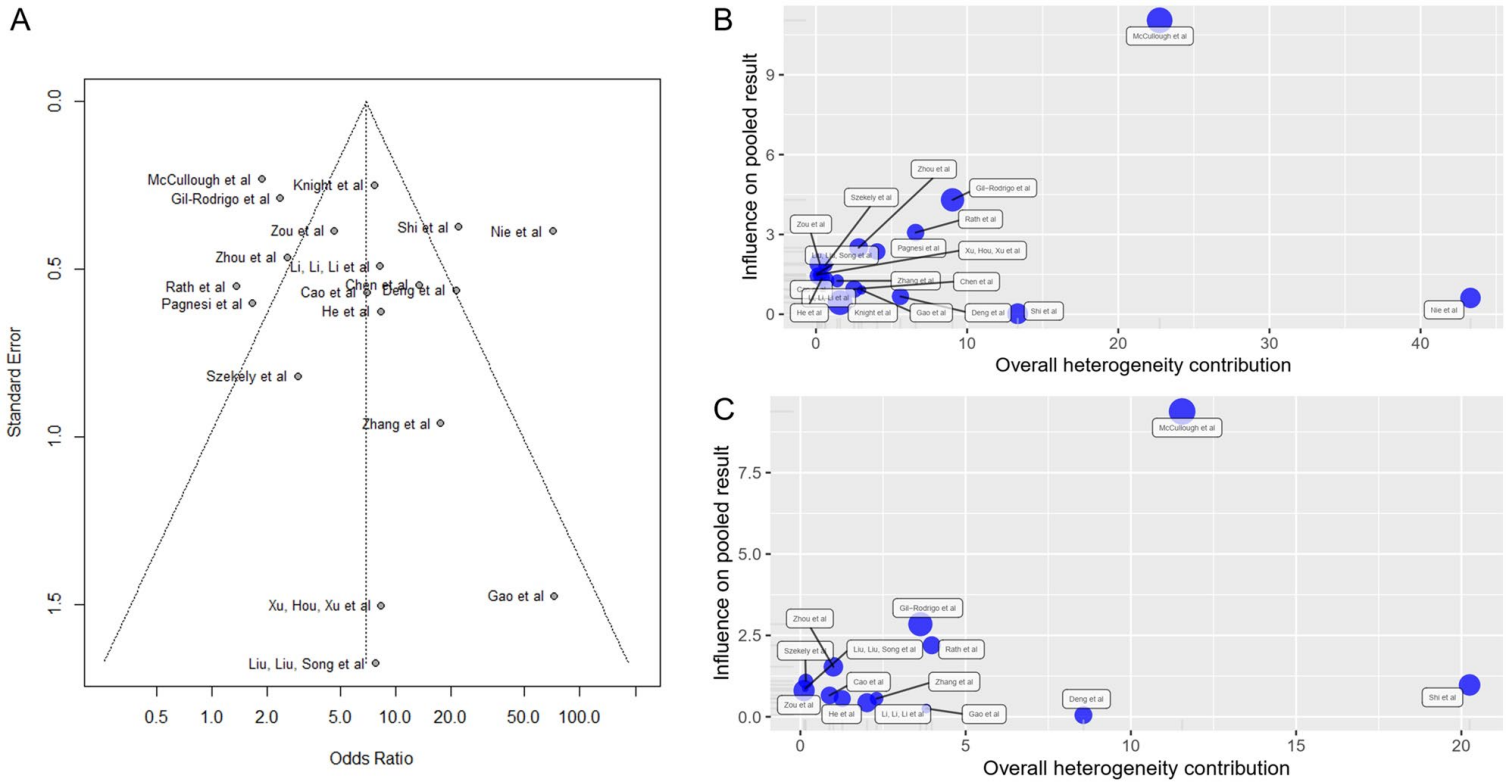
(**A**) Funnel plot of all included studies show a relative paucity of studies with smaller sample sizes and lesser odds ratios (lower-left region). (**B**) Baujat plot of all studies included in the estimation of the pooled odds ratio. (**C**) Baujat plot of all cohort studies included in the estimation of the pooled odds ratio.

A funnel plot of the studies showed little to no publication bias (Fig. 5A). Egger’s Test likewise showed no significant publication bias nor funnel asymmetry, with Intercept = 1.347 [95%-CI (− 1.18; 3.87)], t = 1.045, p = 0.31.

### Heterogeneity and sensitivity analysis

Post-hoc sensitivity analysis was done using methods available in dmetar^12^ and metafor R packages^4^. Among all studies included in the estimation of the pooled odds ratio (OR), McCullough et al. and Nie et al. were identified to be outliers (Supplementary Material 3). Leave-one-out analysis also show that these two studies contribute a significant fraction of the heterogeneity. Excluding Nie et al. would lead to OR of 5.700 [95%-CI (3.535; 9.191)], with I^2^ = 76.6%. Excluding McCullough et al. would lead to OR of 7.545 [95%-CI (4.319; 13.180)], with I^2^ = 81.3%. Baujat diagnostics (Fig. 5B) show that Nie et al. and McCullough et al. contribute to 43.3% and 22.7% of the heterogeneity respectively, totaling to 66% (Supplementary Material 3). Nie et al. used a case–control study design, which has inherent sampling and selection bias. McCullough et al. on the other hand used ECG to define an abnormal cardiac finding. Among all cohort studies included in the estimation of the pooled odds ratio (OR), Shi et al. was identified to be the outlier (Supplementary Material 3). Excluding Shi et al. would lead to OR of 4.725 [95%-CI (2.850; 7.834)], with I^2^ = 67.9. Baujat diagnostics (Fig. 5C) show that Shi et al. and McCullough et al. contribute to 20.2% and 11.6% of the heterogeneity respectively, totaling to 31.8% (Supplementary Material 3). Shi et al. is the only study that used myoglobin as a biomarker for cardiac abnormality.

## Discussion

The substantial heterogeneity among studies precludes any definitive conclusion on the magnitude of risk or odds ratio of severity or mortality associated with any abnormal cardiac finding in any given test. Nevertheless, subgroup analyses of certain cardiac biomarkers—namely, CK, Troponin T, NT-proBNP, as well as Troponin I (if the case–control study by Nie et al. is removed)—show more reliable odds ratios with their nonsignificant heterogeneity. All forest plots show a clear trend towards definite increase in mortality or severity risk among COVID-19 patients exposed to a positive finding in any cardiac abnormality test.

### Implications of the study findings

In another meta-analysis of 35 studies^13^, the pooled frequency of acute cardiac injury among COVID-19 patients was at 25.3%, which is within the bounds of the 95% confidence interval estimated by this study, between 23 and 41%. Hypertension is the most common pre-existing comorbidity in these patients with a pooled frequency of 29.2% (95%-CI 24.7; 33.6%), followed by diabetes with a pooled frequency of 13.5% (95%-CI 11.5; 15.4%)^13^. Overall, fewer than one-fifth of patients had pre-existing cardiovascular diseases, at 12.6% (95%-CI 10.0; 15.2%). The risk of mortality in the presence of acute cardiac injury is increased by nearly 20 times [OR = 19.64; 95%-CI (10.28, 37.53). The heterogeneity of the studies included is also moderately to highly significant; reasons for this were not detailed in the said study. There are currently little to no studies on cardiac abnormalities on only mild and moderate COVID-19 cases as these cases are likely treated on an outpatient basis. Due to overwhelmed health systems in most countries where these studies are done, mild and moderate cases are not given enough attention nor any form of cardiac biomarker screening.

In this study, a pooled odds ratio of 6.87 [95%-CI (3.92; 12.05)] means that, the COVID-19 patients with an abnormal cardiac test are 6.87 times more likely to die or have severe disease than COVID-19 patients without an abnormal cardiac test. At a 5% level of significance, the odds of dying ranges from at least four (4) times to at most twelve (12) times. Nie et al., McCullough et al., and Shi et al. are found to be outlier studies because they used a case–control study design, an ECG finding to define a cardiac abnormality, and serum myoglobin to define a cardiac abnormality respectively. Meanwhile, the Egger’s test showing no significant publication bias means that this pooled odds ratio is sufficiently exhaustive. Newer, larger, longer-term studies are necessary to significantly alter this result.

With regards to severity, persistent symptoms have been reported even after recovery from COVID-19, and this has been linked to cardiac, pulmonary, and neurologic complications^14^. In a study of 143 patients who recovered from COVID-19^14^, 87.4% reported persistence of at least fatigue or dyspnea. In another study of 100 recovered COVID-19 patients^15^, high-sensitivity troponin T (hsTnt) was detectable (3 pg/mL or greater) in 71 patients (71%) and significantly elevated (13.9 pg/mL or greater) in 5 patients (5%). In the same study^15^, 78 patients (78%) had abnormal CMR findings, and endomyocardial biopsy in patients with severe findings revealed active lymphocytic inflammation. These may all contribute to an emerging picture of an emerging epidemic of “COVID-19 associated cardiomyopathy” which may affect survivors who had mild, moderate, severe, or critical COVID-19.

The findings in this meta-analysis may provide an explanation for anecdotal reports of outside-hospital sudden deaths and increasing rates of COVID-19 “recoveries” turning into “deaths”, and more severe disease and more deaths from other comorbid conditions among COVID-19 survivors.

The pooled odds ratio for severity or mortality is but merely a single point estimate of a very fat-tailed risk due to the significant heterogeneity of the included studies, making it necessarily insufficient to give us any definite information for screening efforts^16^. However, there is no doubt on the precautionary principle that should be taken into consideration in implementing policies of recovery and/or follow-up. The risk for severity or mortality across all analyses presented here are asymmetrical and right-skewed. Related distribution of fatalities of pandemic outbreaks in the past 2500 years is strongly fat-tailed^17^. What we are dealing here is an “infectious” form of supposedly the most common cause of death worldwide—cardiac disease that is “infectious”, so to speak.

### Limitations of the study

The authors faced a major challenge in disaggregating the data of each study; hence, studies are pooled together even with different study designs. Some studies have data on prevalence but not on odds ratio. Some studies have multiple tests performed. Thus, only the maximum count of the stated outcome of the study—cardiac abnormalities found through *any* one of the tests—is considered in calculating the pooled prevalence and odds ratio. Consequently, subgroup analyses were performed based on the specific type of test in order to address this limitation; however, the heterogeneity did not fully disappear.

The varying tests and their varying cut-off measures for the definition of a “positive finding” in each individual study likely contributes to a significant portion in the heterogeneity even among the smaller studies. This is an understandable phenomenon given the fact that information is still evolving. Some measures may have reduced validity due to the excessive inflammation in COVID-19, which may cause spuriously high levels of serum biomarkers. Therefore, we propose more studies that will eventually formalize a unified definition or diagnostic criteria for “COVID-19 cardiomyopathy”.

## Conclusion

Despite significant heterogeneity in most comparisons, there is a trend towards definite increase in mortality or severity risk among COVID-19 patients with any cardiac abnormality test.

Due to the high uncertainty in the pooled prevalence and/or incidence of cardiac abnormalities and the unquantifiable magnitude of risk (although an increased risk is certain) for severity or mortality among COVID-19 patients, much more long-term prognostic studies are needed to check for the long-term complications of COVID-19 and formalize definitive criteria of “COVID-19 associated cardiomyopathy”. By defining clear criteria, or by defining a specific test for the detection of any cardiac abnormality, the magnitude of risk can be better measured. Long-term prognostic studies using a defined criteria of “COVID-19 associated cardiomyopathy” on recovered patients should be done.

## Supplementary Information

Supplementary Information 1.

Supplementary Information 2.

Supplementary Information 3.

Supplementary Information 4.
