# Supplementary material for: Prevalence and prognostic associations of cardiac abnormalities among hospitalized patients with COVID-19: a systematic review and meta-analysis
Source: Sci Rep. 2021 Apr 19;11:8449. doi: 10.1038/s41598-021-87961-x (PMC8055982; doi:10.1038/s41598-021-87961-x)
Supplement: Supplementary file 1 — Supplementary Information 1. [file 41598_2021_87961_MOESM1_ESM.pdf]

Prevalence and Prognostic Associations of Cardiac Abnormalities Among Hospitalized Patients with COVID-19 – A Systematic Review and Meta-Analysis

Louie F. Dy1,4*, Ryan C.V. Lintao1, Cynthia P. Cordero2, Ian Theodore G. Cabaluna3 and Leonila F. Dans2,3
1College of Medicine, University of the Philippines Manila, Manila 1000 Philippines
2Department of Clinical Epidemiology, College of Medicine, University of the Philippines Manila, Manila 1000 Philippines
3Asia Pacific Center for Evidence-Based Healthcare, Manila 1000 Philippines
4University of the Philippines COVID-19 Pandemic Response Team, University of the Philippines Resilience Institute, Manila, Philippines
*Corresponding author, E-mail: lfdy@up.edu.ph

SM1_Included-Articles.csv

Key	Item Type	Publicatior	Author	Title	Publicatior	ISBN	ISSN	DOI	Url	Abstract N	Date	Date Adde	Date Modi	Access Dat	Pages	Num Page:	Issue	Volume	Number O
N2DYA5FN	journalArti	2020	Cao, Jiatiar	Myocardia	Theranostics		1838-7640	10.7150/thno.47980		Introductic	2020	########	########		9663-9673		21	10	
6HG8CMV	journalArti	2020	Chen, Cher	Analysis of	Chinese Journal of Ca		0253-3758	10.3760/cı	http://rs.yi	Objective T	3/6/2020	########	########	########	E008-E008		0	48	
63YFU7BC	journalArti	2020	Cummings	Epidemiolo	Lancet (London, Engla		1474-547X	10.1016/S0140-6736(		BACKGROU	6/6/2020	########	########		1763-1770		10239	395	
3A783NZY	journalArti	2020	Deng, Qing	Suspected	International journal c		1874-1754	10.1016/j.ijcard.2020		BACKGROU	########	########	########		116-121			311	
UIYK76XZ	journalArti	2020	Gao, Lei; Ji	Prognostic	Respiratory research		1465-993X	10.1186/s12931-020-		BACKGROU	########	########	########		83		1	21	
CPVLJFBH	journalArti	2020	Ge, Heng;	Cardiac Str	medRxiv			10.1101/20	http://med	BACKGROU	1/1/2020	########	########		2020.05.12.20095885				
PI8NNFHY	journalArti	2020	Gil-Rodrigo	Analysis of	Emergencias : revista		2386-5857	1137-6821		OBJECTIVE	2020	########	########		233-241		4	32	
7FFMUQPT	journalArti	2020	Huang, Ma	Clinical Cha	The American journal		1538-2990	10.1016/j.amjms.2020		BACKGROU	2020-08	########	########		120-128		2	360	
5PSB25H3	journalArti	2020	Knight Dar	Myocardia	Circulation			10.1161/C	https://www.ahajouri		########	########	########	########	1120-1122		11	142	
5UVBEYTP	journalArti	2020	Li, Yuman;	The preval	Intensive Care Medici		1432-1238	10.1007/s0	https://doi.org/10.10		8/7/2020	########	########	########					
EJ24K7FH	journalArti	2020	liu, youbin	Clinical fea	medRxiv			10.1101/20	http://med	Aims To ex	1/1/2020	########	########		2020.03.31.20047142				
F4NTQP5J	journalArti	2020	McCulloug	Electrocar	Journal of cardiac failu		1532-8414	10.1016/j.cardfail.202		BACKGROU	2020-07	########	########		626-632		7	26	
CT9JGVK2	journalArti	2020	Nie Shao-F	Cardiac Tro	Circulation			10.1161/C	https://www.ahajouri		########	########	########	########	608-610		6	142	
IKKK3BZX	journalArti	2020	Pagnesi, M	Pulmonary	Heart (British Cardiac		1468-201X	10.1136/heartjnl-2020		OBJECTIVE	2020-09	########	########		1324-1331		17	106	
WIJ97WX6	journalArti	2020	Rath, Dom	Impaired c	Clinical Research in Ca		1861-0692	10.1007/s0	https://doi	COVID-19 i	########	########	########	########					
SLBSCJPR	journalArti	2020	Shi, Shaob	Characteri	European Heart Journ		0195-668X	10.1093/eu	https://aca	AbstractAi	6/7/2020	########	########	########	2070-2079		22	41	
DQDZLY8Z	journalArti	2020	Szekely Yis	Spectrum c	Circulation			10.1161/C	https://ww	Backgroun	########	########	########	########	342-353		4	142	
5VG8C53I	journalArti	2020	Xu Huayan	Clinical Cha	Journal of the American Heart As			10.1161/JA	https://ww	Backgroun	########	########	########	########	e016807		18	9	
BT6NX84R	journalArti	2020	Xw, He; Js,	[Impact of	Zhonghua xin xue Gua		0253-3758	10.3760/cı	https://eui	Europe PM	6/1/2020	########	########	########	456-460		6	48	
AR5YVHEZ	journalArti	2020	Zhang, Jiar	Myocardia	medRxiv			10.1101/20	https://ww	<p>Backgr	5/9/2020	########	########	########	2020.05.06.20068882				
Y5YJGW9V	journalArti	2020	Zhou, Fei;	Clinical cou	The Lancet		0140-6736	10.1016/S0	http://ww	Backgroun	########	########	########	########	1054-1062		10229	395	
DPINYNJ9	journalArti	2020	Zou, Xiaoji	Acute Phys	Critical care medicine		1530-0293	10.1097/CCM.000000		OBJECTIVE	2020-08	########	########		e657-e665		8	48	
TKXXAVQZ	journalArti	2020	汪, 成; 黎,	新型冠状	中国体外循环杂志					目的 研究	########	########	########				3	18	
C37GY8M(	journalArti	2020	洪, 仲思;	对比分析	新型冠状病毒感染及		1007-3949			目的 回顾	########	########	########				4	28	

Journal Ab	Short Title	Series	Series Nun	Series Text	Series Title	Publisher	Place	Language	Rights	Type	Archive	Archive Lo	Library Cat	Call Numb	Extra	Notes	File Attach	Link Attach	Manual Ta
Theranostics								eng	© The author(s).						PMID: 32863952 PM		C:\Users\Louie Floren		Aged; Fem
								ch	info: Chinese Medical Association and Chine				rs.yiigle.com		Number: 00 Publisher		C:\Users\Louie Florendo Dy\Zote		
Lancet								eng	Copyright © 2020 Elsevier Ltd. All rights reserved.						PMID: 32442528 PM		C:\Users\Louie Floren		Adult; Age
Int J Cardiol								eng	Copyright © 2020 Elsevier B.V. All rights reserved.						PMID: 32291207 PM		C:\Users\Louie Floren		Adult; Fem
Respir Res								eng							PMID: 32293449 PM		C:\Users\Louie Floren		Adult; Age
																	C:\Users\Louie Florendo Dy\Zote		
Emergencias								spa							Place: Spain PMID: 32		C:\Users\Louie Floren		Adult; Age
Am J Med Sci								eng	Copyright © 2020 Southern Society for Clinical Investigation. Publ						Place: United States P		C:\Users\Louie Floren		Adult; Age
Circulation													ahajournals.org (Atyp		Publisher: American H		C:\Users\Louie Florendo Dy\Zote		
Intensive Care Med								en					Springer Link				C:\Users\Louie Florendo Dy\Zote		
																	C:\Users\Louie Florendo Dy\Zote		
J Card Fail								eng	Copyright © 2020 Elsevier Inc. All rights reserved.						PMID: 32544622 PM		C:\Users\Louie Floren		Aged; Age
Circulation													ahajournals.org (Atyp		Publisher: American H		C:\Users\Louie Florendo Dy\Zote		
Heart								eng	© Author(s) (or their employer(s)) 2020. No				commercial re-use. Se		Place: England PMID:		C:\Users\Louie Floren		Female; Hu
Clin Res Cardiol								en					Springer Link				C:\Users\Louie Florendo Dy\Zote		
Eur Heart J								en					academic.oup.com		Publisher: Oxford Aca		C:\Users\Louie Florendo Dy\Zote		
Circulation													ahajournals.org (Atyp		Publisher: American H		C:\Users\Louie Florendo Dy\Zote		
Journal of the American Heart Association													ahajournals.org (Atyp		Publisher: American H		C:\Users\Louie Florendo Dy\Zote		
Zhonghua Xin Xue Guan Bing Za Zhi								English					europepmc.org		PMID: 32171190		C:\Users\Louie Florendo Dy\Zote		
								en	© 2020, Posted by Cold Spring Harbor Labor				www.medrxiv.org		Publisher: Cold Spring		C:\Users\Louie Florendo Dy\Zote		
The Lancet	Clinical course and risk factors for mortality of adult inpatients with COVID-1							en					ScienceDirect				C:\Users\Louie Florendo Dy\Zote		
Crit Care Med								eng							PMID: 32697506 PM		C:\Users\Louie Floren		Adult; Age
																	C:\Users\Louie Florendo Dy\Zote		
																	C:\Users\Louie Florendo Dy\Zote		

Automatic Editor	Series Edit	Translator	Contributo	Attorney A	Book Auth	Cast Meml	Commente	Composer	Cosponsor	Counsel	Interviewe	Producer	Recipient	Reviewed	Scriptwrite	Words By	Guest	Number

ale; Humans; Male; Middle Aged; Pandemics; Retrospective Studies; Prognosis; China; Cohort Studies; Predictive Value of Tests; *COVID-19; Hospitalization; Troponin I/*blood; *in-hospital fatality; *myocardial injury; *Trop

ro\storage\VS467UVL\Chen et al. - 2020 - Analysis of myocardial injury in patients with COV.pdf

d; Aged, 80 and over; Female; Humans; Male; Middle Aged; Pandemics; Young Adult; Age Factors; Betacoronavirus; Fibrin Fibrinogen Degradation Products/analysis; Risk Factors; Prospective Studies; Biomarkers/blood; Res

ale; Humans; Male; Middle Aged; Pandemics; Retrospective Studies; Treatment Outcome; Comorbidity; Prognosis; China; Cohort Studies; Biomarkers/blood; Risk Assessment; *COVID-19; Severity of Illness Index; Survival A

d; Female; Humans; Male; Middle Aged; Retrospective Studies; Prognosis; Betacoronavirus; Clinical Laboratory Techniques; Risk Factors; COVID-19; SARS-CoV-2; *Pandemics; *Hospital Mortality; Predictive Value of Tests; M

ro\storage\4NHDV8E2\Ge et al. - 2020 - Cardiac Structural and Functional Characteristics .pdf

d; Aged, 80 and over; Female; Humans; Male; Middle Aged; Pandemics; Respiration, Artificial/statistics & numerical data; Comorbidity; Prognosis; Young Adult; Adolescent; Age Factors; *Betacoronavirus; Age Distribution; C

d; Aged, 80 and over; Female; Humans; Male; Middle Aged; Retrospective Studies; China; *Betacoronavirus; *Pandemics; *COVID-19; *Coronavirus Infections/blood/mortality/therapy; *Pneumonia, Viral/blood/mortality/t

ro\storage\7ZQYYANK\Knight Daniel S. et al. - 2020 - Covid-19.pdf

ro\storage\QVUXS434\Li et al. - 2020 - The prevalence, risk factors and outcome of cardia.pdf

ro\storage\26U694TJ\liu et al. - 2020 - Clinical features and outcomes of 2019 novel coron.pdf

d, 80 and over; Female; Humans; Male; Middle Aged; Pandemics; Retrospective Studies; Cohort Studies; *Betacoronavirus; *mortality; *COVID-19; *myocardial injury; Hospital Mortality/trends; *electrocardiography; Bundl

ro\storage\TLYPIHN5\Nie Shao-Fang et al. - 2020 - Cardiac Troponin I Is an Independent Predictor for.pdf; C:\Users\Louie Florendo Dy\Zotero\storage\5FTQPRRW\CIRCULATIONAHA.120.html

umans; Male; Middle Aged; Comorbidity; *Pandemics; Hospitalization/statistics & numerical data; Severity of Illness Index; Italy/epidemiology; Prevalence; Correlation of Data; Betacoronavirus/*isolation & purification; Out

ro\storage\J47JAY4S\Rath et al. - 2020 - Impaired cardiac function is associated with morta.pdf

ro\storage\YWVEMSPU\Shi et al. - 2020 - Characteristics and clinical significance of myoca.pdf; C:\Users\Louie Florendo Dy\Zotero\storage\TSLN85R9\5835730.html

ro\storage\SPLXV6JA\Szekely Yishay et al. - 2020 - Spectrum of Cardiac Manifestations in COVID-19.pdf; C:\Users\Louie Florendo Dy\Zotero\storage\5LL5QI2K\CIRCULATIONAHA.120.html

ro\storage\XGFQ7DM5\Xu Huayan et al. - 2020 - Clinical Characteristics and Risk Factors of Cardi.pdf; C:\Users\Louie Florendo Dy\Zotero\storage\KUG9FSJT\JAHA.120.html

ro\storage\K99UIZKA\Xw et al. - 2020 - [Impact of complicated myocardial injury on the cl.pdf

ro\storage\Y4IQJL2F\Zhang et al. - 2020 - Myocardial characteristics as the prognosis for CO.pdf; C:\Users\Louie Florendo Dy\Zotero\storage\WRSXSH83\2020.05.06.html

ro\storage\YTXS5TKH\S0140673620305663.html; C:\Users\Louie Florendo Dy\Zotero\storage\3FUF8MJ6\Zhou et al. - 2020 - Clinical course and risk factors for mortality of .pdf

d; China/epidemiology; Female; Humans; Intensive Care Units/statistics & numerical data; Male; Middle Aged; Pandemics; Retrospective Studies; *Hospital Mortality; Predictive Value of Tests; Hospitalization/statistics & nu

ro\storage\AYQD95BZ\汪 et al. - 2020 - 新型冠状病毒肺炎患者心肌损伤的临床分析.pdf

ro\storage\SBZI43B6\洪 et al. - 2020 - 对比分析 18 例重症 危重症新型冠状病毒肺炎心肌损伤患者临床特征.pdf

Edition Running Ti Scale Medium Artwork Si Filing Date Applicatio Assignee Issuing Aut Country Meeting N Conferenc Court References Reporter Legal Statu Priority Nu Programm Version System

ponin I; Cardiomyopathies/blood/*etiology; Coronavirus Infections/blood/*complications/mortality; Pneumonia, Viral/blood/*complications/mortality

piration, Artificial; Hospitalization; Hospital Mortality; Respiratory Distress Syndrome, Adult/virology; Proportional Hazards Models; New York City/epidemiology; Critical Illness/epidemiology; Interleukin-6/blood; Coronavi

nalysis; Hospitalization/*statistics & numerical data; *Cause of Death; *Cardiac marker; *Myocardial injury; *Myocarditis; *Novel coronavirus; Coronary Angiography/methods; Coronavirus Infections/*diagnosis/*epidemic

Mortality; *Coronavirus Infections/diagnosis/mortality; *Pneumonia, Viral/diagnosis/mortality; Natriuretic Peptide, Brain/*analysis; NT-proBNT; Outcome; Peptide Fragments/*analysis; Reference Values

Cardiovascular Diseases/epidemiology; Prospective Studies; *SARS-CoV-2; *COVID-19; Hospital Mortality; Sex Distribution; Logistic Models; Odds Ratio; *Comorbidity; Child; Child, Preschool; Infant; Infant, Newborn; Spain/

herapy; *Critical care; *Disease progression; *Respiratory Distress Syndrome, Adult/blood/mortality/therapy; *Severe patients

e-Branch Block/diagnosis/*mortality/physiopathology; Coronavirus Infections/diagnosis/*mortality/physiopathology; Electrocardiography/methods/*mortality; Heart Failure/diagnosis/*mortality/physiopathology; Pneum

tcome Assessment, Health Care; *Coronavirus Infections/complications/diagnosis/epidemiology/physiopathology; *echocardiography; *Hypertension, Pulmonary/diagnosis/epidemiology/etiology; *Pneumonia, Viral/comp

merical data; Logistic Models; Proportional Hazards Models; *Cause of Death; ROC Curve; Causality; Coronavirus Infections/*diagnosis/*epidemiology/therapy; Pneumonia, Viral/*diagnosis/*epidemiology/therapy; Organ

Code	Code Num Section	Session	Committee History	Legislative Body

rus Infections/*epidemiology/mortality/*therapy; Pneumonia, Viral/*epidemiology/mortality/*therapy

logy; Echocardiography, Doppler/methods; Hospitals, University; Myocardial Infarction/diagnosis/*epidemiology/therapy; Myocarditis/diagnosis/*epidemiology/therapy; Pneumonia, Viral/*diagnosis/*epidemiology

epidemiology; Emergency Service, Hospital/*statistics & numerical data; *Características clínicas; *Clinical characteristics; *Comorbilidades; *Coronavirus disease 2019 (COVID-19); *Emergency health services; *Servicios de

onia, Viral/diagnosis/*mortality/physiopathology

lications/diagnosis/epidemiology/physiopathology; *pulmonary vascular disease; *Ventricular Dysfunction, Right/diagnosis/epidemiology/etiology; Echocardiography/methods

Dysfunction Scores; Hospitals, Urban; APACHE; Severe Acute Respiratory Syndrome/diagnosis/*mortality/therapy; Survivors/statistics & numerical data

urgencias; *Severe acute respiratory syndrome coronavirus 2 (SARS-CoV-2); *Symptom Assessment; Coronavirus Infections/*diagnosis/*epidemiology/mortality/therapy; Intubation, Intratracheal/statistics & numerical da

ıta; Neoplasms/epidemiology; Obesity/complications; Pneumonia, Viral/*diagnosis/*epidemiology/mortality/therapy; Respiration Disorders/epide
