# Supplementary material for: Prevalence and prognostic associations of cardiac abnormalities among hospitalized patients with COVID-19: a systematic review and meta-analysis
Source: Sci Rep. 2021 Apr 19;11:8449. doi: 10.1038/s41598-021-87961-x (PMC8055982; doi:10.1038/s41598-021-87961-x)
Supplement: Supplementary file 2 — Supplementary Information 2. [file 41598_2021_87961_MOESM2_ESM.pdf]

Prevalence and Prognostic Associations of Cardiac Abnormalities Among Hospitalized Patients with COVID-19 – A Systematic Review and Meta-Analysis

Louie F. Dy1,4*, Ryan C.V. Lintao1, Cynthia P. Cordero2, Ian Theodore G. Cabaluna3 and Leonila F. Dans2,3
1College of Medicine, University of the Philippines Manila, Manila 1000 Philippines
2Department of Clinical Epidemiology, College of Medicine, University of the Philippines Manila, Manila 1000 Philippines
3Asia Pacific Center for Evidence-Based Healthcare, Manila 1000 Philippines
4University of the Philippines COVID-19 Pandemic Response Team, University of the Philippines Resilience Institute, Manila, Philippines
*Corresponding author, E-mail: lfdy@up.edu.ph

SM1_Search-Strategy_step-by-step.txt

Supplementary Material 1. Search Strategy

PubMed (includes MEDLINE)

A. Using PubMed Advanced Search

1. Search Strategy

#1 ("2019 nCoV" OR "2019nCoV" OR "2019-nCoV" OR "COVID 19" OR "COVID19" OR "COVID-19" OR "new coronavirus" OR "novel coronavirus" OR (Wuhan AND coronavirus) OR (Wuhan AND pneumonia) OR "SARS-CoV" OR "SARS-CoV-2" OR "SARS CoV-2")
#2 #1 AND ("troponin" OR "cardiac" OR "myocardial" OR "TnI" OR "TnT" OR "cardiovascular" OR "heart" OR "CMR" OR "cardiac MRI" OR "cardiac magnetic resonance imaging" OR "echocardiography" OR "BNP"

OR "brain natiuretic peptide" OR "NT-proBNP")
#3 #2 AND (("observational study"[Publication Type]) OR ("clinical trial"[Publication Type]))
#4 #2 AND ("observational study"[Publication Type])

Result:
- 113 studies for #3
- 85 studies for #4.

#3 was chosen as the initial field of screening.

2. Titles were screened.
- 45 Eligible Studies
- 68 Excluded: 63 Not Relevant, 5 Protocol Summaries

3. Abstracts were screened.
- 36 Eligible Studies
- 9 Excluded: 3 Not Relevant, 3 No Comparator, 3 Study Design

Result: 36 eligible studies.

EMBASE

Currently, there is no institutional access for EMBASE.

Google Scholar

1. Free text search was done in Google Scholar using the following keywords:

"COVID-19 cardiac echocardiography"
"COVID-19 cardiac troponin"
"COVID-19 cardiac MRI"

Result: 120 records found.
Duplicates: 14
Working Field: 106

2. Titles were screened.
- 21 Included
- 85 Excluded: 7 Letter, Editorial, Review; 3 Study Design, 89 Not Relevant

3. Abstracts were screened.

- 20 Included
- 1 Excluded: 1 No Comparator

Cochrane Library

1. Go to "https://www.cochranelibrary.com/advanced-search".
2. The following query was used:

("COVID-19" OR "coronavirus" OR "2019-nCoV") AND ("cardiac" OR "cardiovascular" OR "myocardial" OR "troponin" OR "echocardiography" OR "BNP" OR "NT-proBNP" OR "cardiac MRI" OR "CMR")

3. We found 2 Cochrane Reviews, 104 Trials, and 158 Reviews.
4. Titles were screened.
- 5 Eligible Articles

5. Abstracts were screened.
- 1 Eligible Article
- Excluded: 4 Study Design

BioRxiv and MedRxiv

Attempt #1

1. Go to "https://www.medrxiv.org/".
2. Click "COVID-19 SARS-CoV-2 preprints from medRxiv and bioRxiv" below the search box.
3. No results found.

Attempt #2

1. Go to Advanced Search.
2. Set date from 12/01/2019 to 09/24/2020.
3. Select medRxiv.
4. Select Cardiovascular Medicine.
5. Under Full Text / Abstract / Title, enter "COVID-19, coronavirus, 2019-nCoV".
6. Click the radio button "any". Click Search.
7. Found 65 results, saved as "medRxiv.ris".
8. No results were found for Cardiovascular Medicine under bioRxiv.
9. Opened the file using Zotero, a bibliography manager software.
10. Titles were screened.
- 13 Eligible Articles
- 52 Excluded: 3 Different Study Population, 13 Systematic Reviews, 36 Not Relevant
11. Abstracts were screened.
- 9 Eligible Articles

- 4 Excluded: 3 Different Comparator, 1 Different Outcome

9 articles are found to be eligible for full text screening.

CNKI
1. Go to "https://oversea.cnki.net/index/"
2. On the upper right corner beside the search box, type "新型冠状病毒" at the main search box. 40595 results.
3. Click "TR-clinical research" on the left side column top.
4. Click "Confirm" on the left side. 1447 results.
5. Under Subject > Main, click "心机损伤" (myocardial injury).
6. Click "Confirm" on the left side. 12 results.
7. Results saved as "CNKI-20200924160418216.xls".
8. Titles were screened. 10 are found to be eligible.
- 1 Duplicate
- 1 Not Relevant based on Title
9. Abstracts were screened.
- 3 Eligible Studies
- 7 Excluded: 1 Not Relevant, 6 Systematic Reviews

Result: 3 Eligible Studies.

[1]刘欢,马小峰,谭剑凯,黄柯,周琴怡,姜志胜.COVID-19重型患者心肌损伤及心脏功能变化的初步观察[J].中国动脉硬化杂志,2020,28(04):285-289.
[2]洪仲思,郑新春,杨小月,曲秀娟,黄曦.对比分析18例重症/危重症新型冠状病毒肺炎心肌损伤患者临床特征[J].中国动脉硬化杂志,2020,28(04):290-295.
[3]鲁亦凡,顾伟,李志忠,张京梅.新型冠状病毒肺炎合并心肌损

伤的研究现状[J].心肺血管病杂志,2020,39(07):773-775.

ChinaXiv

Attempt #1

1. Go to "http://www.chinaxiv.org/user/search.htm".
2. On the upper right corner beside the search box, click "Advanced Search".
3. On the filters below, click "Medicine, Pharmacy", then make sure only "Clinical Medicine" and "Pharmacy" tick boxes are checked.
4. Type "COVID-19, coronavirus, 2019-nCoV" as the search query. Select "All Fields" from the drop down box beside it.
5. Set the date to be from 2019-12-01 to 2020-09-24.
6. Click "Search".
7. Results saved as "ChinaXiv Attempt 1.txt".
8. Titles were screened. No eligible studies.

Result: No identified eligible studies.

ChinXiv Attempt #2

1. Go to "http://www.chinaxiv.org/user/search.htm".
2. Enter "新型冠状病毒" at the main search box.
3. Results (17) saved as "ChinaXiv Attempt 2.txt".
4. Titles were screened. No eligible studies.

Result: No identified eligible studies.
