# Supplementary material for: Prevalence and prognostic associations of cardiac abnormalities among hospitalized patients with COVID-19: a systematic review and meta-analysis
Source: Sci Rep. 2021 Apr 19;11:8449. doi: 10.1038/s41598-021-87961-x (PMC8055982; doi:10.1038/s41598-021-87961-x)
Supplement: Supplementary file 3 — Supplementary Information 3. [file 41598_2021_87961_MOESM3_ESM.pdf]

Prevalence and Prognostic Associations of Cardiac Abnormalities Among Hospitalized Patients with COVID-19 – A Systematic Review and Meta-Analysis

Louie F. Dy[1,4*], Ryan C.V. Lintao[1], Cynthia P. Cordero[2], Ian Theodore G. Cabaluna[3] and Leonila F. Dans[2,3]
[1]College of Medicine, University of the Philippines Manila, Manila 1000 Philippines
[2]Department of Clinical Epidemiology, College of Medicine, University of the Philippines Manila, Manila 1000 Philippines
[3]Asia Pacific Center for Evidence-Based Healthcare, Manila 1000 Philippines
[4]University of the Philippines COVID-19 Pandemic Response Team, University of the Philippines Resilience Institute, Manila, Philippines
*Corresponding author, E-mail: lfdy@up.edu.ph

SM2_Risk-of-Bias-Assessment.docx

Supplementary Material 2. Risk of Bias Assessments

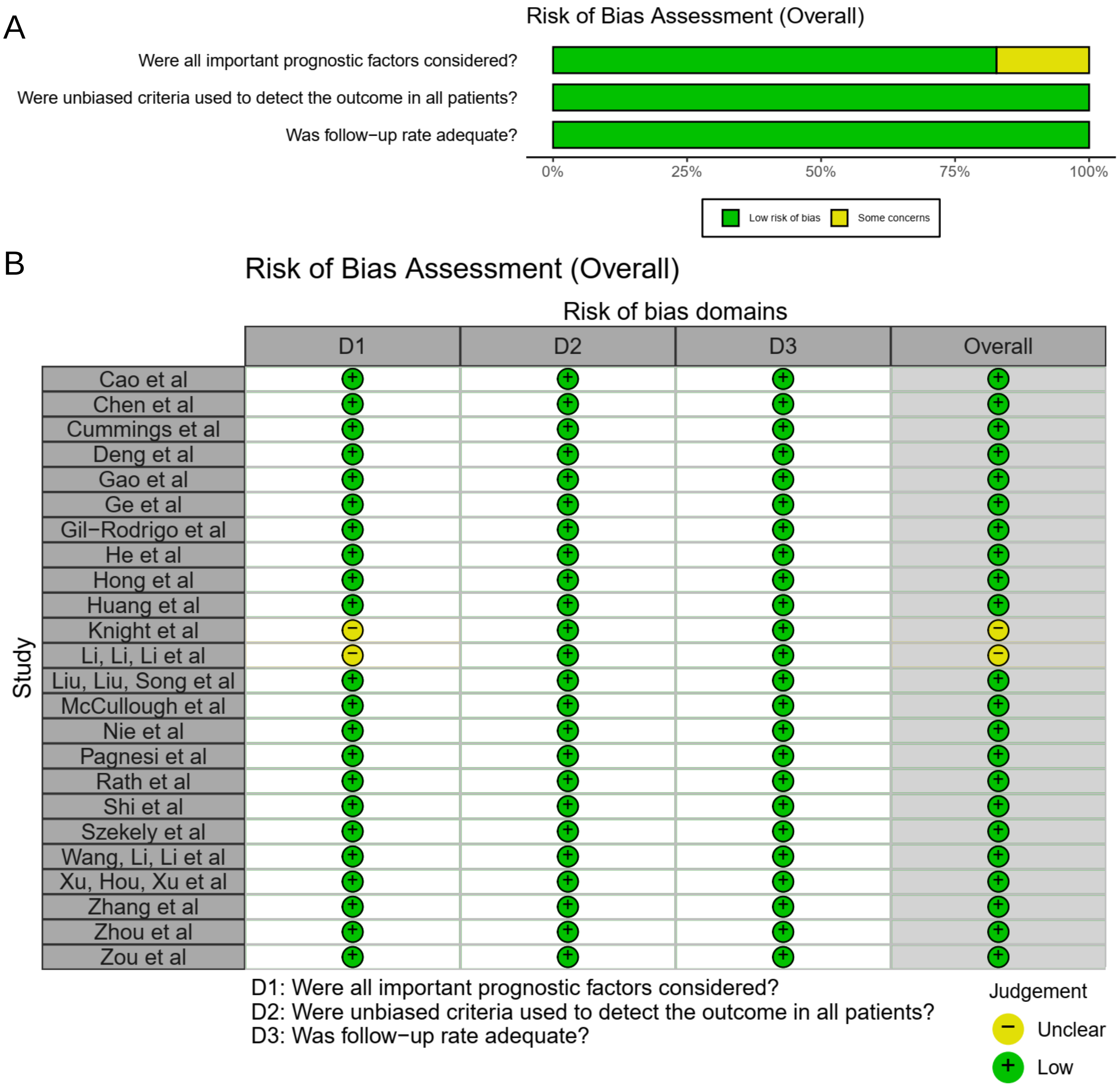

Supplemental Figure 1. (A) Summary findings of the risk of bias assessment of the 24 studies. The left column shows the signaling questions used in the assessment. (B) Risk of bias assessment domains for each of the 24 studies.
