# Supplementary material for: Prevalence and prognostic associations of cardiac abnormalities among hospitalized patients with COVID-19: a systematic review and meta-analysis
Source: Sci Rep. 2021 Apr 19;11:8449. doi: 10.1038/s41598-021-87961-x (PMC8055982; doi:10.1038/s41598-021-87961-x)
Supplement: Supplementary file 4 — Supplementary Information 4. [file 41598_2021_87961_MOESM4_ESM.pdf]

Prevalence and Prognostic Associations of Cardiac Abnormalities Among Hospitalized Patients with COVID-19 – A Systematic Review and Meta-Analysis

Louie F. Dy[1,4*], Ryan C.V. Lintao[1], Cynthia P. Cordero[2], Ian Theodore G. Cabaluna[3] and Leonila F. Dans[2,3]
[1]College of Medicine, University of the Philippines Manila, Manila 1000 Philippines
[2]Department of Clinical Epidemiology, College of Medicine, University of the Philippines Manila, Manila 1000 Philippines
[3]Asia Pacific Center for Evidence-Based Healthcare, Manila 1000 Philippines
[4]University of the Philippines COVID-19 Pandemic Response Team, University of the Philippines Resilience Institute, Manila, Philippines
*Corresponding author, E-mail: lfdy@up.edu.ph

SM3_Sensitivity-Analysis.pptx

Sensitivity Analysis

Analyses Performed: Briefer

- Outlier Remover
  - An automatic function that removes outliers
- Leave-One-Out Analysis
  - Remove one study and see its effect on heterogeneity
- Influence Diagnostics
  - Several metrics

Pooled OR

Pooled OR of All Studies

Study	Exposure Events	Exposure Total	Control Events	Control Total	OR	95%−CI	Weight
**Study Design = Cohort**							
Cao et al	8	45	6	199	6.95	[2.28; 21.22]	6.4%
Deng et al	26	42	5	70	21.13	[7.02; 63.61]	6.4%
Gao et al	18	30	0	24	72.52	[4.03; 1305.46]	2.1%
Gil−Rodrigo et al	18	80	101	920	2.35	[1.34; 4.14]	8.7%
Li, Li, Li et al	15	40	8	117	8.18	[3.12; 21.39]	7.0%
Liu, Liu, Song et al	1	10	0	24	7.74	[0.29; 207.08]	1.7%
McCullough et al	37	219	53	537	1.86	[1.18; 2.92]	0.0%
Rath et al	10	69	6	54	1.36	[0.46; 4.00]	6.5%
Shi et al	53	182	9	489	21.91	[10.53; 45.60]	8.0%
Szekely et al	8	60	2	40	2.92	[0.59; 14.55]	4.6%
He et al	18	24	8	30	8.25	[2.42; 28.17]	5.9%
Zhang et al	14	16	4	14	17.50	[2.67; 114.85]	3.8%
Zhou et al	11	22	41	146	2.56	[1.03; 6.36]	7.2%
Zou et al	25	42	27	112	4.63	[2.18; 9.83]	7.9%
**Random effects model**		881		2776	6.40	[3.56; 11.48]	76.3%
Heterogeneity: $I^2 = 73\%$, $\tau^2 = 0.7395$, $p < 0.01$							
**Study Design = Cross−Sectional**							
Chen et al	19	47	5	103	13.30	[4.56; 38.81]	6.6%
Pagnesi et al	4	29	15	171	1.66	[0.51; 5.42]	6.1%
**Random effects model**		76		274	4.78	[0.62; 36.75]	12.7%
Heterogeneity: $I^2 = 85\%$, $\tau^2 = 1.8368$, $p = 0.01$							
**Study Design = Case−Control**							
Knight et al	239	586	20	242	7.65	[4.70; 12.43]	9.0%
Nie et al	91	103	20	208	71.28	[33.40; 152.15]	0.0%
Xu, Hou, Xu et al	4	55	0	47	8.30	[0.44; 158.32]	2.0%
**Random effects model**		744		497	7.66	[4.74; 12.38]	11.0%
Heterogeneity: $I^2 = 0\%$, $\tau^2 = 0$, $p = 0.96$							
**Random effects model**		**1701**		**3547**	**6.24**	**[3.92; 9.95]**	**100.0%**
Heterogeneity: $I^2 = 70\%$, $\tau^2 = 0.5641$, $p < 0.01$							
Residual heterogeneity: $I^2 = 73\%$, $p < 0.01$							

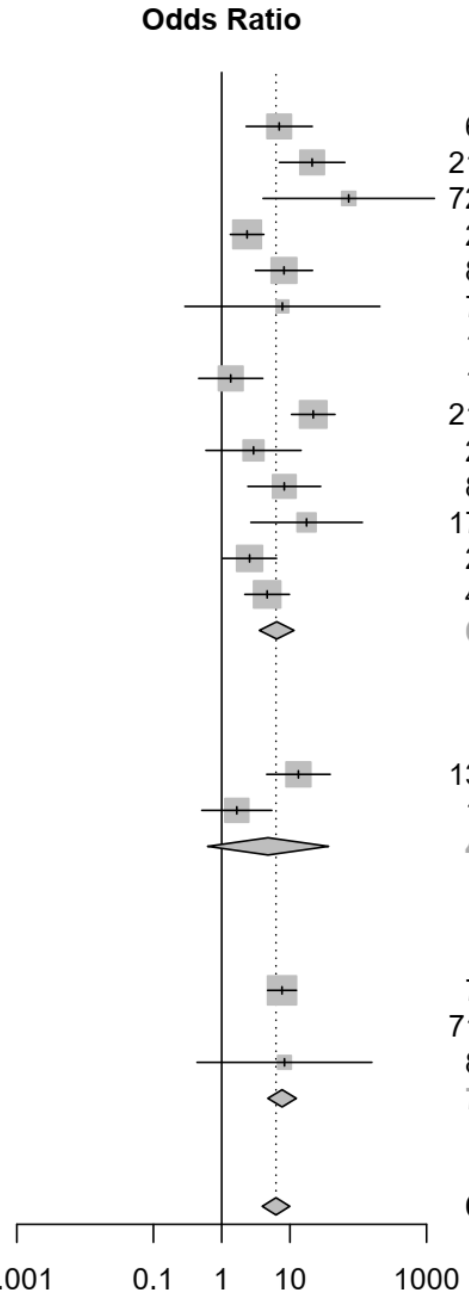

```
Identified outliers (random-effects model)
------------------------------------------
"McCullough et al", "Nie et al"

Results with outliers removed
-----------------------------
                              OR              95%-CI %W(random)    Study Design exclude
Cao et al                 6.9550 [ 2.2797;   21.2184]       6.4          Cohort
Deng et al               21.1250 [ 7.0152;   63.6139]       6.4          Cohort
Gao et al                72.5200 [ 4.0286; 1305.4562]       2.1          Cohort
Gil-Rodrigo et al         2.3542 [ 1.3394;    4.1380]       8.7          Cohort
Li, Li, Li et al          8.1750 [ 3.1243;   21.3904]       7.0          Cohort
Liu, Liu, Song et al      7.7368 [ 0.2891;  207.0783]       1.7          Cohort
McCullough et al          1.8565 [ 1.1800;    2.9208]       0.0          Cohort       *
Rath et al                1.3559 [ 0.4598;    3.9986]       6.5          Cohort
Shi et al                21.9121 [10.5295;   45.5999]       8.0          Cohort
Szekely et al             2.9231 [ 0.5872;   14.5500]       4.6          Cohort
He et al                  8.2500 [ 2.4158;   28.1741]       5.9          Cohort
Zhang et al              17.5000 [ 2.6666;  114.8459]       3.8          Cohort
Zhou et al                2.5610 [ 1.0305;    6.3645]       7.2          Cohort
Zou et al                 4.6296 [ 2.1802;    9.8309]       7.9          Cohort
Chen et al               13.3000 [ 4.5578;   38.8102]       6.6 Cross-Sectional
Pagnesi et al             1.6640 [ 0.5108;    5.4207]       6.1 Cross-Sectional
Knight et al              7.6452 [ 4.7009;   12.4338]       9.0    Case-Control
Nie et al                71.2833 [33.3974;  152.1468]       0.0    Case-Control       *
Xu, Hou, Xu et al         8.3010 [ 0.4352;  158.3169]       2.0    Case-Control

Number of studies combined: k = 17
```

```
                                OR             95%-CI     z  p-value
Random effects model 6.2434 [3.9177; 9.9498] 7.70 < 0.0001

Quantifying heterogeneity:
 tau^2 = 0.5641; tau = 0.7511; I^2 = 69.5% [49.9%; 81.5%]; H = 1.81 [1.41; 2.32]

Quantifying residual heterogeneity:
 I^2 = 72.5% [54.0%; 83.6%]; H = 1.91 [1.47; 2.47]

Test of heterogeneity:
     Q d.f.  p-value
 52.54   16 < 0.0001

Results for subgroups (random effects model):
                                  k     OR              95%-CI  tau^2    tau     Q   I^2
Study Design = Cohort            13 6.3957 [3.5619; 11.4841] 0.7395 0.8599 44.43 73.0%
Study Design = Cross-Sectional    2 4.7777 [0.6211; 36.7506] 1.8368 1.3553  6.55 84.7%
Study Design = Case-Control       2 7.6619 [4.7418; 12.3804]      0      0  0.00  0.0%

Test for subgroup differences (random effects model):
                     Q d.f. p-value
Between groups    0.36    2  0.8346

Details on meta-analytical method:
- Mantel-Haenszel method
- DerSimonian-Laird estimator for tau^2
- Mantel-Haenszel estimator used in calculation of Q and tau^2 (like RevMan 5)
- Continuity correction of 0.5 in studies with zero cell frequencies
```

Influence Analysis of Pooled OR

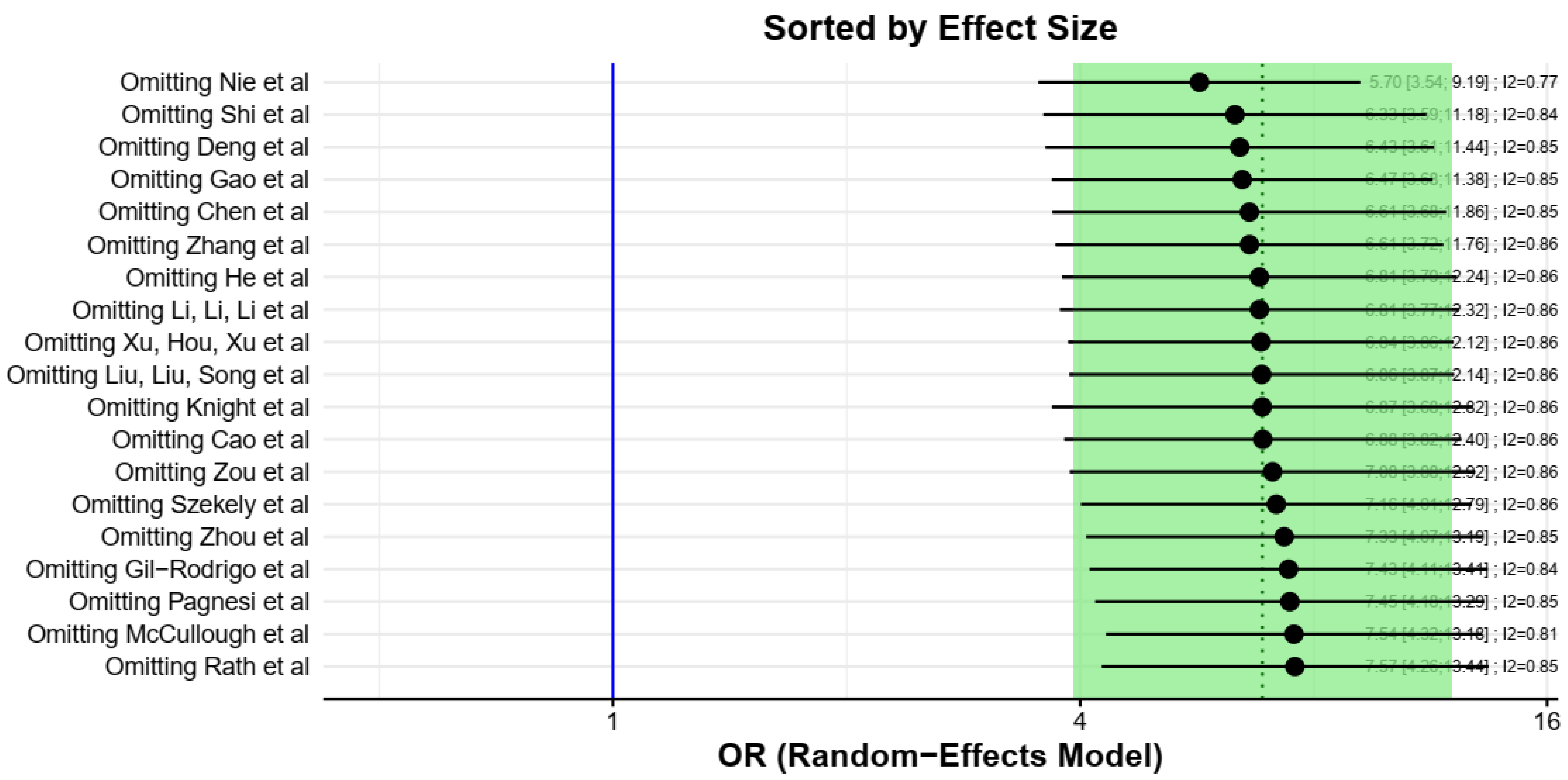
Sorted by Effect Size
Omitting Nie et al
Omitting Shi et al
Omitting Deng et al
Omitting Gao et al
Omitting Chen et al
Omitting Zhang et al
Omitting He et al
Omitting Li, Li, Li et al
Omitting Xu, Hou, Xu et al
Omitting Liu, Liu, Song et al
Omitting Knight et al
Omitting Cao et al
Omitting Zou et al
Omitting Szekely et al
Omitting Zhou et al
Omitting Gil−Rodrigo et al
Omitting Pagnesi et al
Omitting McCullough et al
Omitting Rath et al
5.70 [3.54; 9.19] ; I2=0.77
6.33 [3.59;11.18] ; I2=0.84
6.43 [3.61;11.44] ; I2=0.85
6.47 [3.68;11.38] ; I2=0.85
6.61 [3.68;11.86] ; I2=0.85
6.61 [3.72;11.76] ; I2=0.86
6.81 [3.79;12.24] ; I2=0.86
6.81 [3.77;12.32] ; I2=0.86
6.84 [3.86;12.12] ; I2=0.86
6.86 [3.87;12.14] ; I2=0.86
6.87 [3.68;12.82] ; I2=0.86
6.88 [3.82;12.40] ; I2=0.86
7.08 [3.88;12.92] ; I2=0.86
7.16 [4.01;12.79] ; I2=0.86
7.33 [4.07;13.19] ; I2=0.85
7.43 [4.11;13.41] ; I2=0.84
7.45 [4.18;13.29] ; I2=0.85
7.54 [4.32;13.18] ; I2=0.81
7.57 [4.26;13.44] ; I2=0.85
1
4
16
OR (Random−Effects Model)

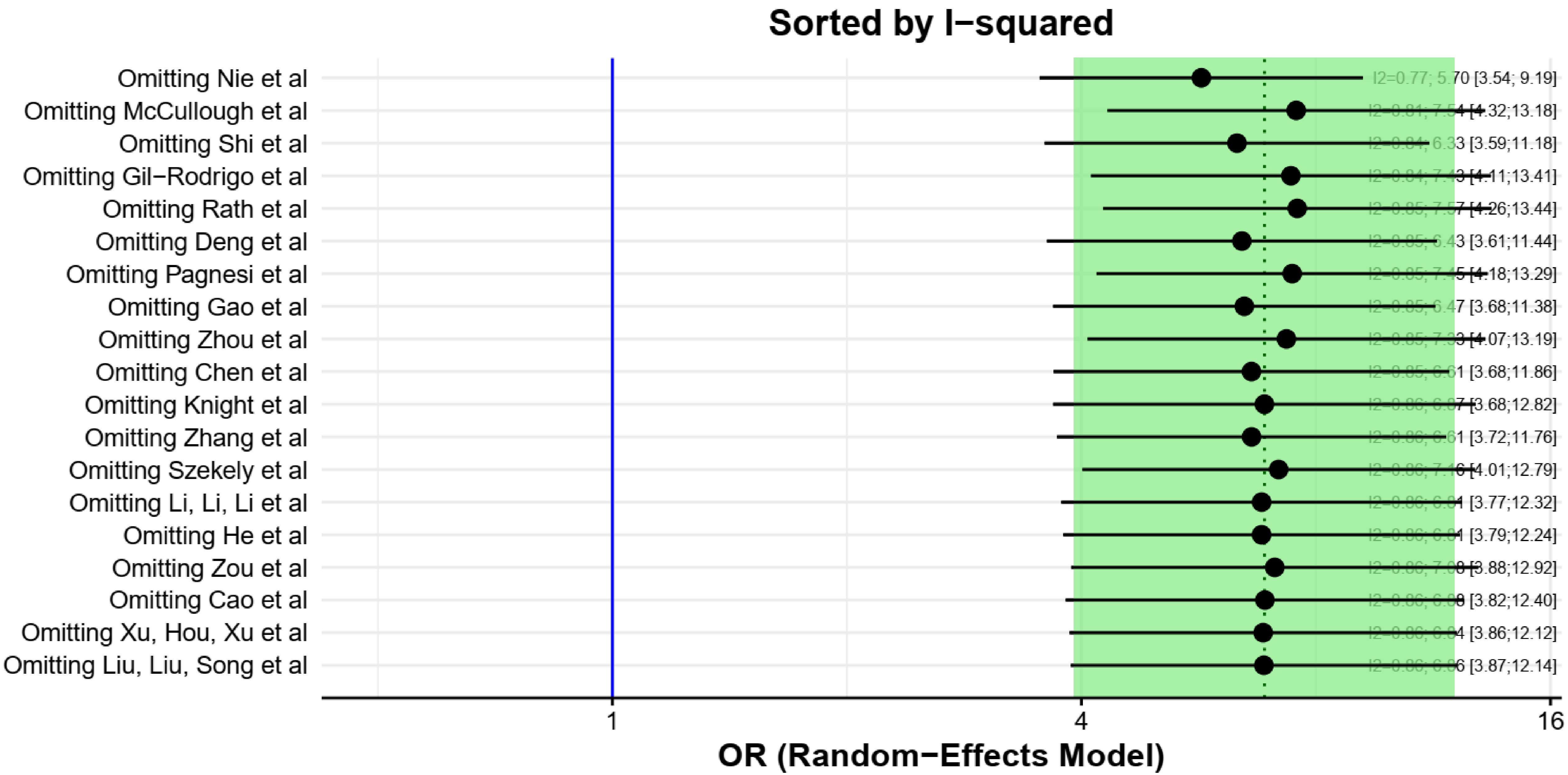
Sorted by I−squared
Omitting Nie et al
Omitting McCullough et al
Omitting Shi et al
Omitting Gil−Rodrigo et al
Omitting Rath et al
Omitting Deng et al
Omitting Pagnesi et al
Omitting Gao et al
Omitting Zhou et al
Omitting Chen et al
Omitting Knight et al
Omitting Zhang et al
Omitting Szekely et al
Omitting Li, Li, Li et al
Omitting He et al
Omitting Zou et al
Omitting Cao et al
Omitting Xu, Hou, Xu et al
Omitting Liu, Liu, Song et al
I2=0.77; 5.70 [3.54; 9.19]
[3.59;11.18]
[3.61;11.44]
[3.68;11.38]
[3.68;11.86]
[3.68;12.82]
[3.72;11.76]
[3.77;12.32]
[3.79;12.24]
[3.82;12.40]
[3.86;12.12]
[3.87;12.14]
1
4
16
OR (Random−Effects Model)

Leave-One-Out Analysis (Sorted by I2)

	Effect	LLCI	ULCI	I2
Omitting Nie et al	5.700	3.535	9.191	0.766
Omitting McCullough et al	7.545	4.319	13.180	0.813
Omitting Shi et al	6.333	3.587	11.181	0.838
Omitting Gil-Rodrigo et al	7.427	4.114	13.408	0.843
Omitting Rath et al	7.567	4.262	13.436	0.849
Omitting Deng et al	6.426	3.611	11.435	0.851
Omitting Pagnesi et al	7.455	4.183	13.288	0.852
Omitting Gao et al	6.471	3.679	11.385	0.854
Omitting Zhou et al	7.329	4.072	13.194	0.854
Omitting Chen et al	6.610	3.684	11.861	0.855
Omitting Knight et al	6.869	3.679	12.824	0.855
Omitting Zhang et al	6.613	3.719	11.757	0.856
Omitting Li, Li, Li et al	6.812	3.768	12.316	0.857
Omitting Szekely et al	7.162	4.010	12.793	0.857
Omitting He et al	6.811	3.790	12.241	0.857
Omitting Zou et al	7.081	3.880	12.922	0.857
Omitting Cao et al	6.879	3.817	12.399	0.858
Omitting Liu, Liu, Song et al	6.857	3.875	12.137	0.858
Omitting Xu, Hou, Xu et al	6.843	3.862	12.124	0.858

Note how Nie et al (red dot) is an outlier. (Influence Plot)

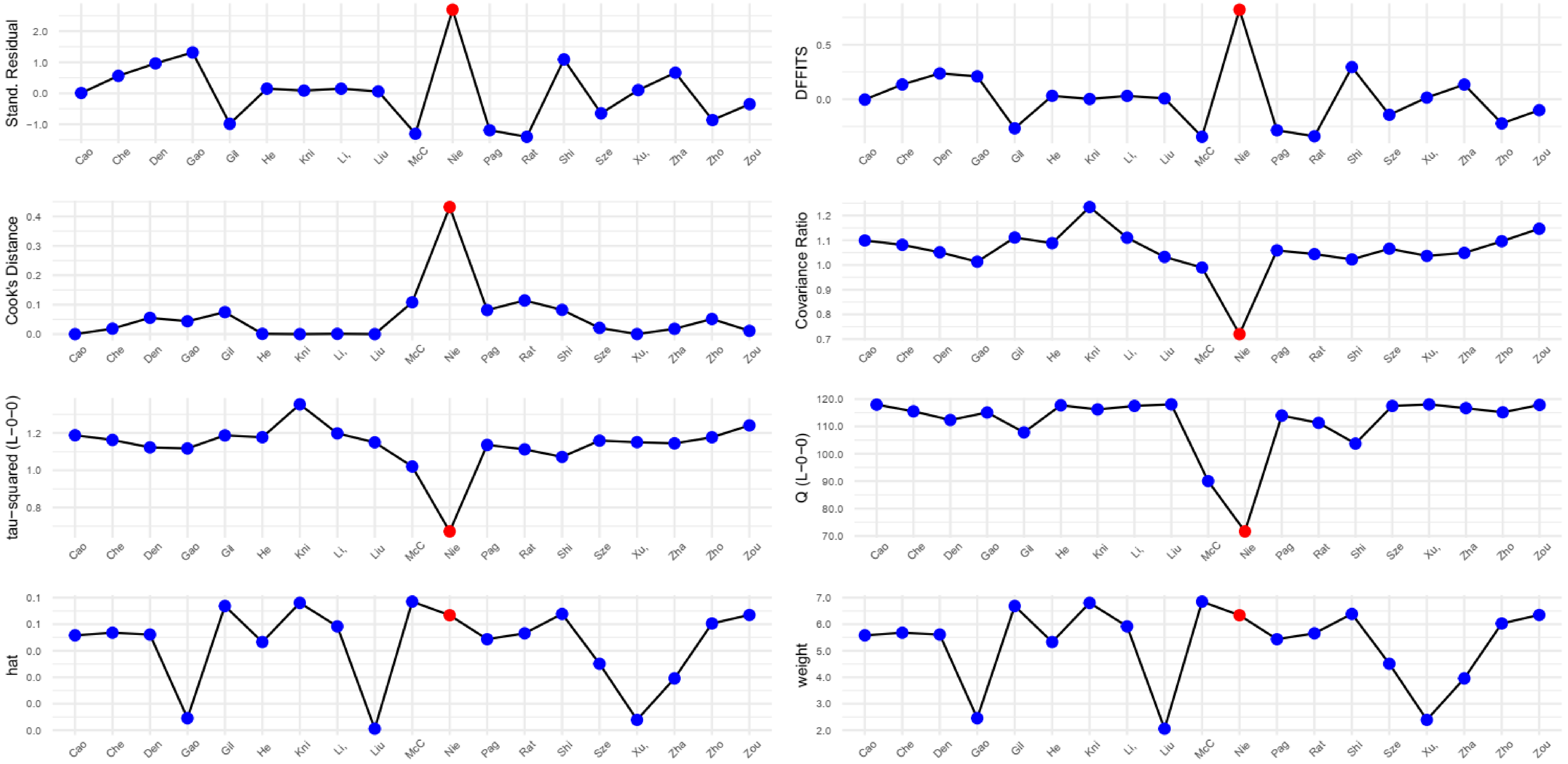

Influence Diagnostics

----

	rstudent	dffits	cook.d	cov.r	QE.del	hat	weight	infl
Omitting Cao et al	0.009	-0.004	0.000	1.099	117.958	0.056	5.579	
Omitting Deng et al	0.964	0.236	0.055	1.051	112.339	0.056	5.608	
Omitting Gao et al	1.316	0.209	0.044	1.013	115.076	0.025	2.458	
Omitting Gil-Rodrigo et al	-0.985	-0.268	0.075	1.111	107.814	0.067	6.684	
Omitting Li, Li, Li et al	0.148	0.030	0.001	1.110	117.485	0.059	5.916	
Omitting Liu, Liu, Song et al	0.060	0.007	0.000	1.033	118.073	0.021	2.061	
Omitting McCullough et al	-1.305	-0.346	0.108	0.990	90.044	0.068	6.849	
Omitting Rath et al	-1.403	-0.341	0.114	1.044	111.292	0.057	5.654	
Omitting Shi et al	1.091	0.294	0.082	1.022	103.720	0.064	6.385	
Omitting Szekely et al	-0.647	-0.144	0.021	1.066	117.474	0.045	4.507	
Omitting He et al	0.149	0.031	0.001	1.088	117.715	0.053	5.327	
Omitting Zhang et al	0.664	0.134	0.018	1.049	116.682	0.040	3.957	
Omitting Zhou et al	-0.863	-0.223	0.051	1.096	115.146	0.060	6.026	
Omitting Zou et al	-0.349	-0.101	0.011	1.147	117.852	0.063	6.346	
Omitting Chen et al	0.562	0.135	0.018	1.082	115.506	0.057	5.677	
Omitting Pagnesi et al	-1.191	-0.286	0.082	1.059	113.949	0.054	5.433	
Omitting Knight et al	0.087	0.001	0.000	1.235	116.205	0.068	6.803	
Omitting Nie et al	2.694	0.822	0.432	0.720	71.653	0.063	6.336	*
Omitting Xu, Hou, Xu et al	0.104	0.014	0.000	1.037	118.041	0.024	2.394	

Note how Nie et al and McCullough et al are outliers. (Baujat Plot)

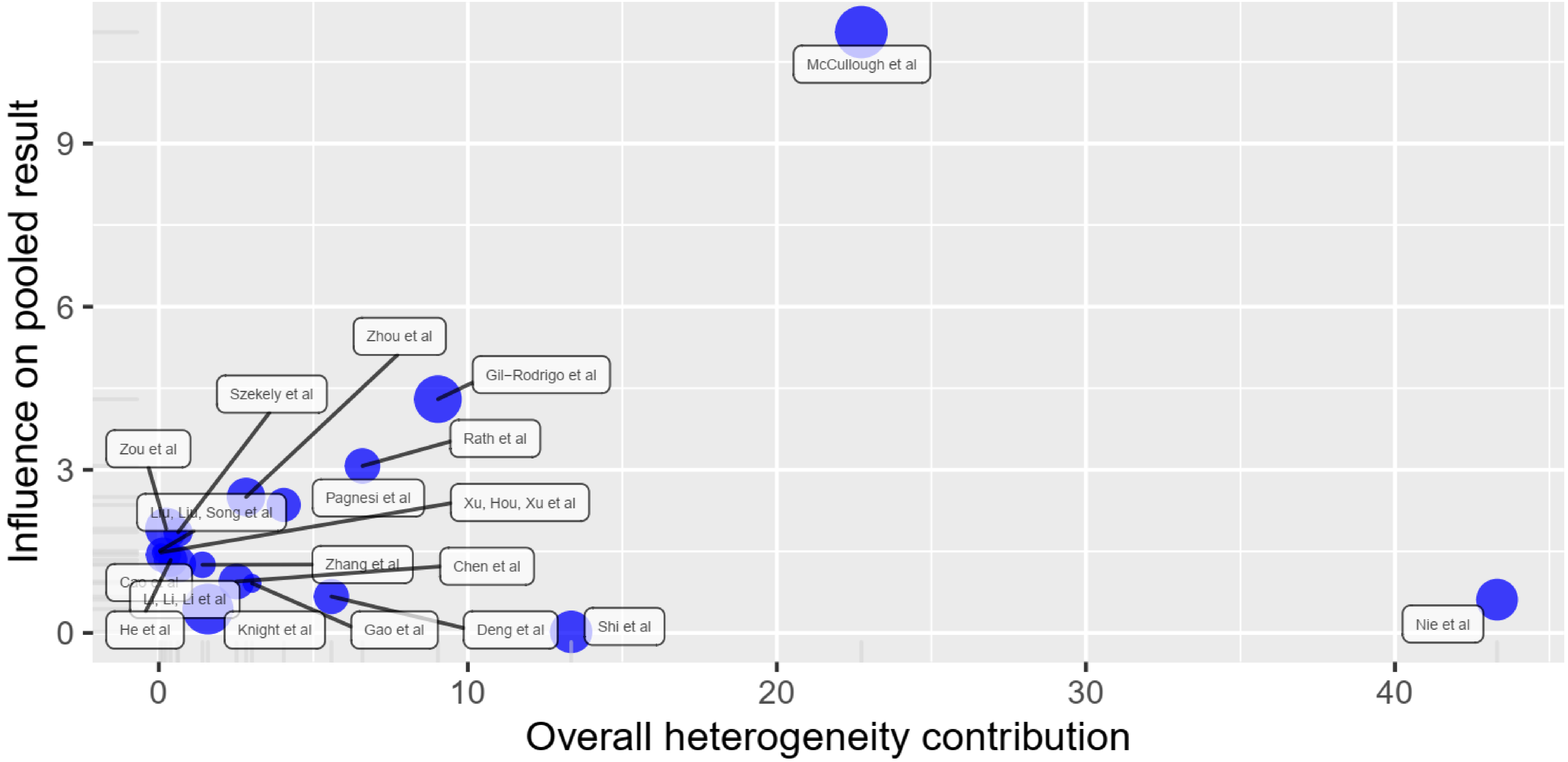

Baujat Diagnostics (sorted by Heterogeneity Contribution)

	HetContrib	InfluenceEffectSize
Omitting Nie et al	43.304	0.613
Omitting McCullough et al	22.733	11.047
Omitting Shi et al	13.345	0.020
Omitting Gil-Rodrigo et al	9.033	4.300
Omitting Rath et al	6.591	3.071
Omitting Deng et al	5.587	0.671
Omitting Pagnesi et al	4.045	2.355
Omitting Gao et al	3.020	0.913
Omitting Zhou et al	2.825	2.501
Omitting Chen et al	2.516	0.946
Omitting Knight et al	1.591	0.440
Omitting Zhang et al	1.413	1.254
Omitting Szekely et al	0.627	1.852
Omitting Li, Li, Li et al	0.600	1.266
Omitting He et al	0.386	1.341
Omitting Zou et al	0.241	1.918
Omitting Cao et al	0.147	1.437
Omitting Xu, Hou, Xu et al	0.069	1.481
Omitting Liu, Liu, Song et al	0.038	1.509

Cohort OR

Pooled OR of Cohort Studies

Study	Exposure Events	Exposure Total	Control Events	Control Total	OR	95%−CI	Weight
Cao et al	8	45	6	199	6.95	[2.28; 21.22]	9.3%
Deng et al	26	42	5	70	21.13	[7.02; 63.61]	9.4%
Gao et al	18	30	0	24	72.52	[4.03; 1305.46]	2.8%
Gil−Rodrigo et al	18	80	101	920	2.35	[1.34; 4.14]	13.4%
Li, Li, Li et al	15	40	8	117	8.18	[3.12; 21.39]	10.4%
Liu, Liu, Song et al	1	10	0	24	7.74	[0.29; 207.08]	2.2%
McCullough et al	37	219	53	537	1.86	[1.18; 2.92]	0.0%
Rath et al	10	69	6	54	1.36	[0.46; 4.00]	9.5%
Shi et al	53	182	9	489	21.91	[10.53; 45.60]	0.0%
Szekely et al	8	60	2	40	2.92	[0.59; 14.55]	6.4%
He et al	18	24	8	30	8.25	[2.42; 28.17]	8.5%
Zhang et al	14	16	4	14	17.50	[2.67; 114.85]	5.3%
Zhou et al	11	22	41	146	2.56	[1.03; 6.36]	10.8%
Zou et al	25	42	27	112	4.63	[2.18; 9.83]	12.0%
**Random effects model**		**881**		**2776**	**5.35**	**[3.15; 9.10]**	**100.0%**
**Prediction interval**						**[1.05; 27.36]**	

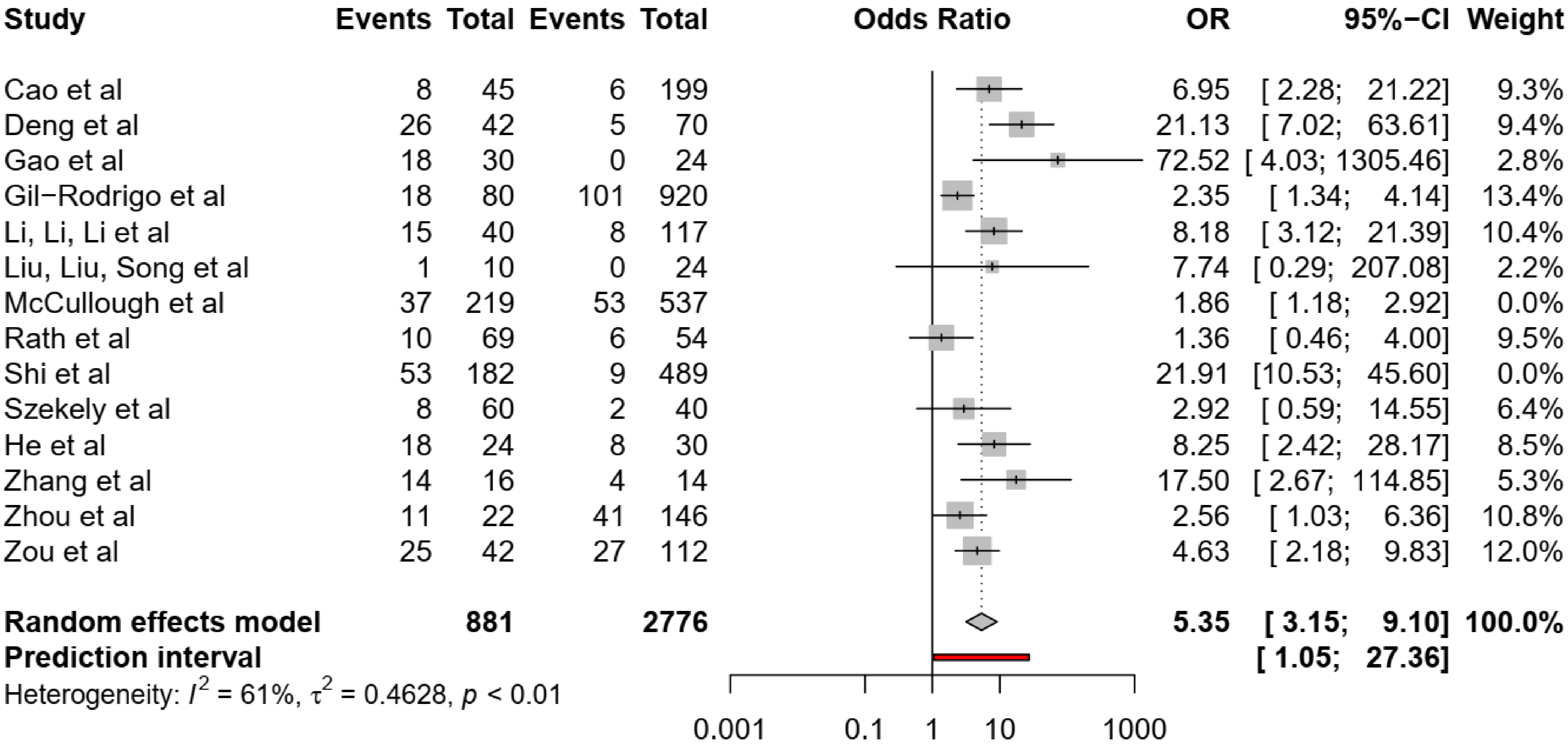

Heterogeneity: $I^2 = 61\%$, $\tau^2 = 0.4628$, $p < 0.01$

Identified outliers (random-effects model)

"McCullough et al", "Shi et al"

Results with outliers removed

	OR	95%-CI	%W(random)	exclude
Cao et al	6.9550	[2.2797; 21.2184]	9.3	
Deng et al	21.1250	[7.0152; 63.6139]	9.4	
Gao et al	72.5200	[4.0286; 1305.4562]	2.8	
Gil-Rodrigo et al	2.3542	[1.3394; 4.1380]	13.4	
Li, Li, Li et al	8.1750	[3.1243; 21.3904]	10.4	
Liu, Liu, Song et al	7.7368	[0.2891; 207.0783]	2.2	
McCullough et al	1.8565	[1.1800; 2.9208]	0.0	*
Rath et al	1.3559	[0.4598; 3.9986]	9.5	
Shi et al	21.9121	[10.5295; 45.5999]	0.0	*
Szekely et al	2.9231	[0.5872; 14.5500]	6.4	
He et al	8.2500	[2.4158; 28.1741]	8.5	
Zhang et al	17.5000	[2.6666; 114.8459]	5.3	
Zhou et al	2.5610	[1.0305; 6.3645]	10.8	
Zou et al	4.6296	[2.1802; 9.8309]	12.0	

Number of studies combined: k = 12

```
                                OR              95%-CI     z  p-value
Random effects model 5.3549 [3.1523;  9.0965] 6.21 < 0.0001
Prediction interval         [1.0480; 27.3614]

Quantifying heterogeneity:
 tau^2 = 0.4628; tau = 0.6803; I^2 = 60.8% [26.3%; 79.1%]; H = 1.60 [1.16; 2.19]

Test of heterogeneity:
     Q d.f. p-value
 28.04   11  0.0032

Details on meta-analytical method:
- Mantel-Haenszel method
- DerSimonian-Laird estimator for tau^2
- Mantel-Haenszel estimator used in calculation of Q and tau^2 (like RevMan 5)
- Continuity correction of 0.5 in studies with zero cell frequencies
```

Influence Analysis of Cohort RR

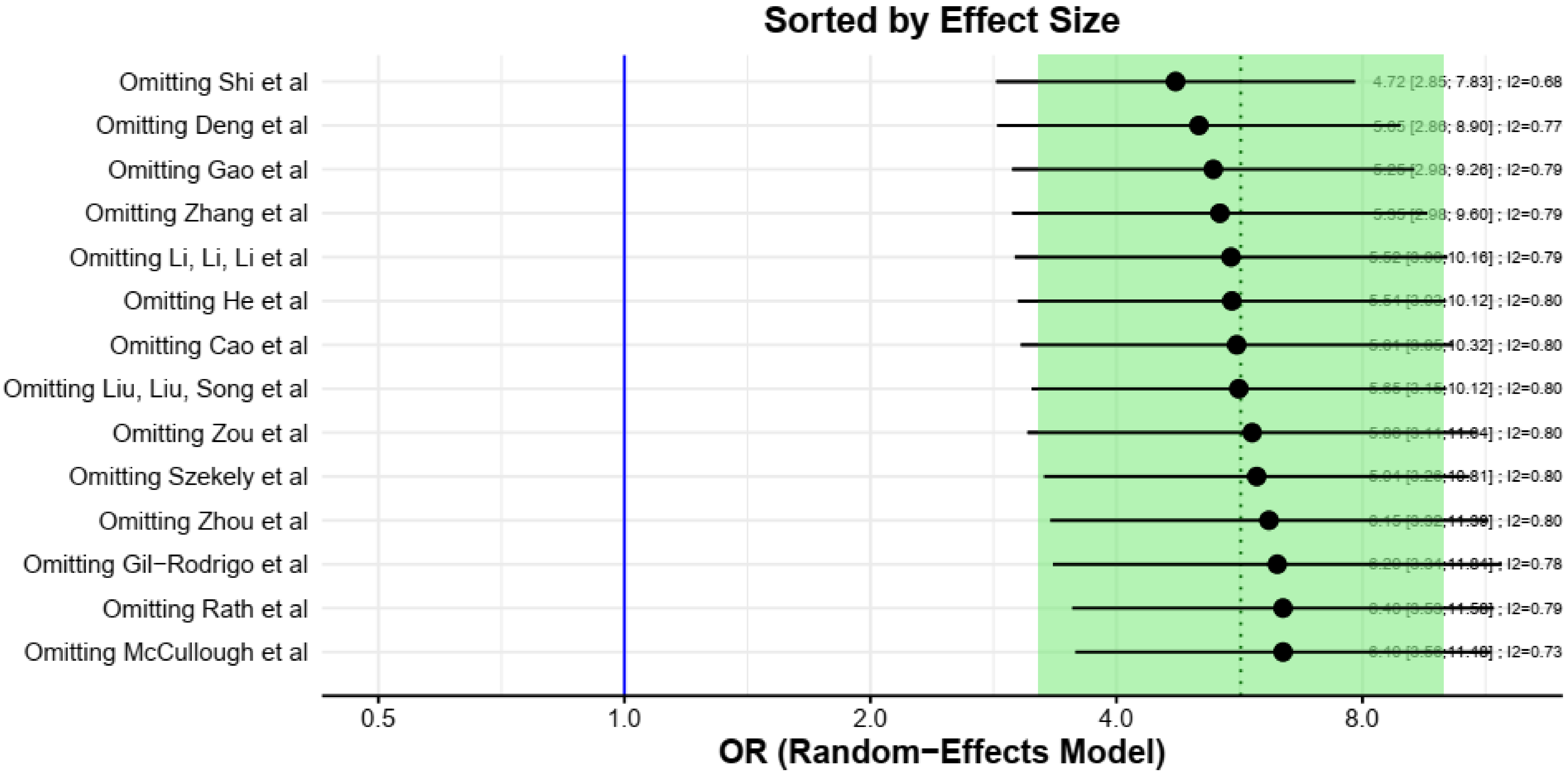
Sorted by Effect Size
Omitting Shi et al
Omitting Deng et al
Omitting Gao et al
Omitting Zhang et al
Omitting Li, Li, Li et al
Omitting He et al
Omitting Cao et al
Omitting Liu, Liu, Song et al
Omitting Zou et al
Omitting Szekely et al
Omitting Zhou et al
Omitting Gil−Rodrigo et al
Omitting Rath et al
Omitting McCullough et al
4.72 [2.85; 7.83] ; I2=0.68
; 8.90] ; I2=0.77
; 9.26] ; I2=0.79
; 9.60] ; I2=0.79
10.16] ; I2=0.79
10.12] ; I2=0.80
10.32] ; I2=0.80
10.12] ; I2=0.80
; I2=0.80
; I2=0.80
; I2=0.80
; I2=0.78
; I2=0.79
; I2=0.73
0.5
1.0
2.0
4.0
8.0
OR (Random−Effects Model)

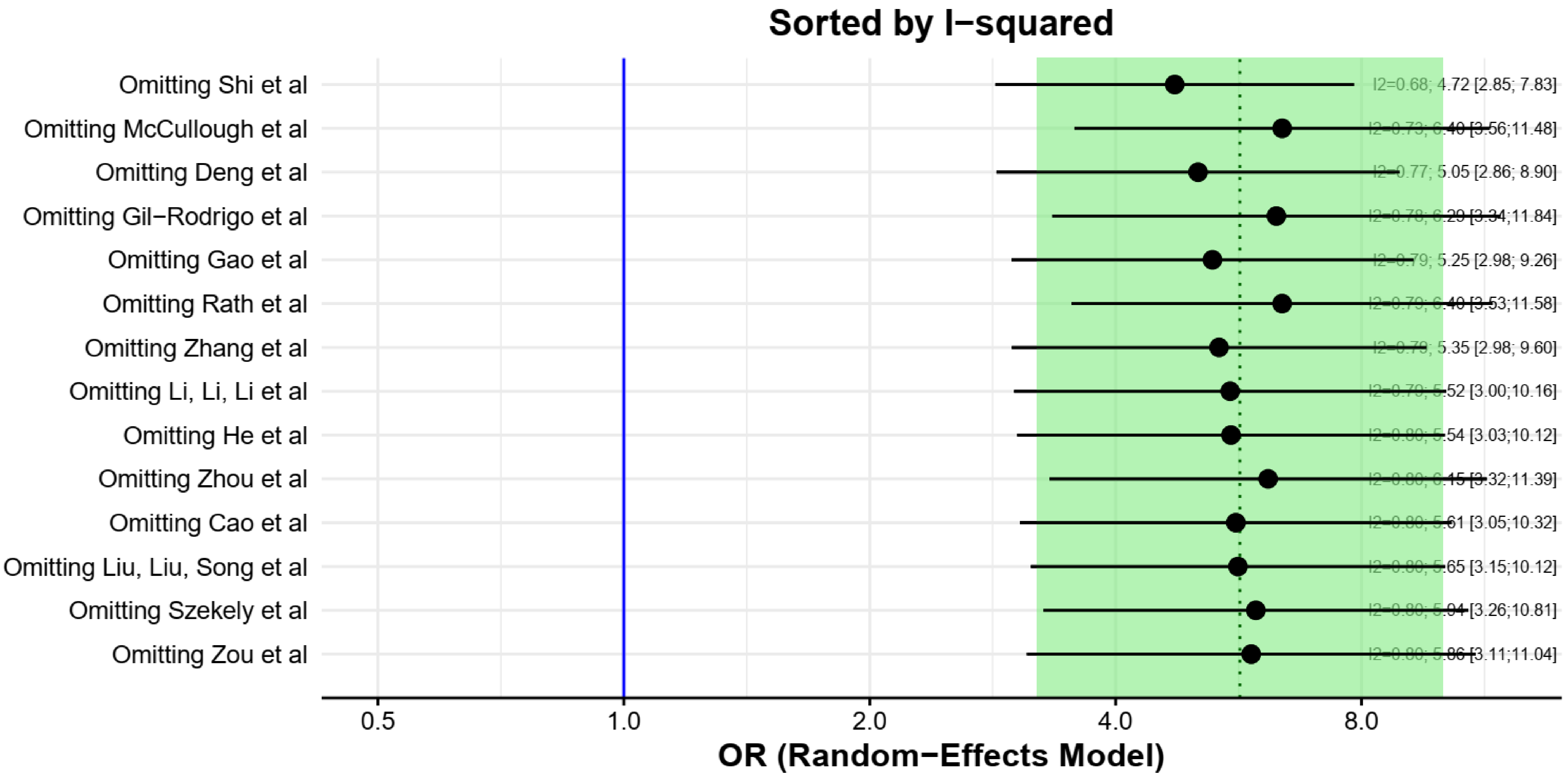
Sorted by I−squared
Omitting Shi et al
Omitting McCullough et al
Omitting Deng et al
Omitting Gil−Rodrigo et al
Omitting Gao et al
Omitting Rath et al
Omitting Zhang et al
Omitting Li, Li, Li et al
Omitting He et al
Omitting Zhou et al
Omitting Cao et al
Omitting Liu, Liu, Song et al
Omitting Szekely et al
Omitting Zou et al
I2=0.68; 4.72 [2.85; 7.83]
I2=0.73; 6.40 [3.56;11.48]
I2=0.77; 5.05 [2.86; 8.90]
I2=0.78; 6.29 [3.34;11.84]
I2=0.79; 5.25 [2.98; 9.26]
I2=0.79; 6.40 [3.53;11.58]
I2=0.79; 5.35 [2.98; 9.60]
I2=0.79; 5.52 [3.00;10.16]
I2=0.80; 5.54 [3.03;10.12]
I2=0.80; 6.15 [3.32;11.39]
I2=0.80; 5.61 [3.05;10.32]
I2=0.80; 5.65 [3.15;10.12]
I2=0.80; 5.94 [3.26;10.81]
I2=0.80; 5.86 [3.11;11.04]
0.5
1.0
2.0
4.0
8.0
OR (Random−Effects Model)

Leave-One-Out Analysis (Sorted by I2)

	Effect	LLCI	ULCI	I2
Omitting Shi et al	4.725	2.850	7.834	0.679
Omitting McCullough et al	6.396	3.562	11.484	0.730
Omitting Deng et al	5.046	2.859	8.905	0.767
Omitting Gil-Rodrigo et al	6.292	3.345	11.838	0.785
Omitting Gao et al	5.255	2.982	9.261	0.787
Omitting Rath et al	6.395	3.532	11.581	0.787
Omitting Zhang et al	5.353	2.984	9.604	0.793
Omitting Li, Li, Li et al	5.524	3.003	10.160	0.795
Omitting He et al	5.537	3.030	10.116	0.797
Omitting Zhou et al	6.147	3.318	11.388	0.798
Omitting Cao et al	5.613	3.054	10.318	0.799
Omitting Liu, Liu, Song et al	5.646	3.149	10.123	0.801
Omitting Szekely et al	5.939	3.261	10.814	0.801
Omitting Zou et al	5.862	3.114	11.038	0.801

Influence plot of all cohort studies

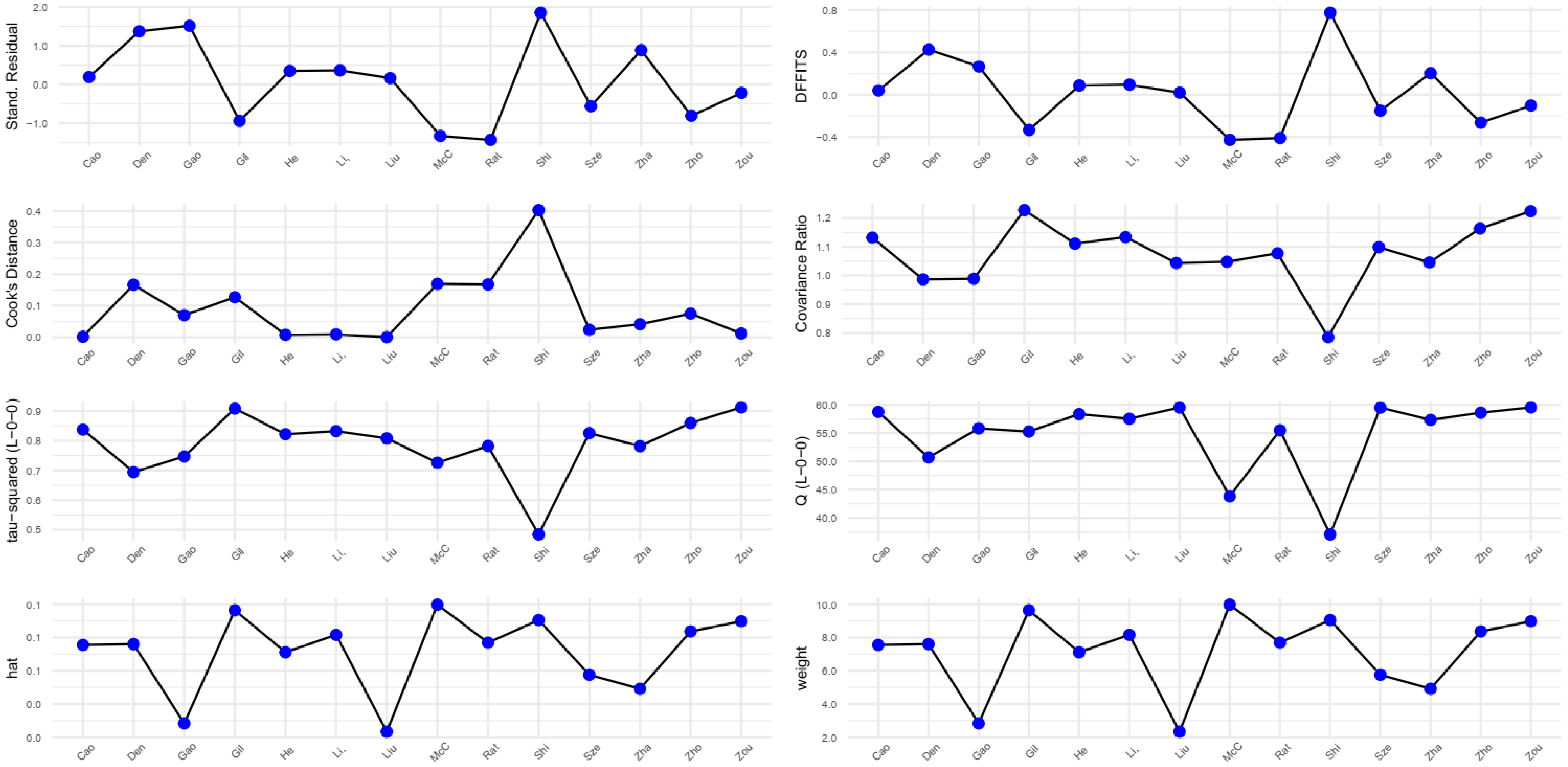

Influence Diagnostics

	rstudent	dffits	cook.d	cov.r	QE.del	hat	weight	infl
Omitting Cao et al	0.193	0.040	0.002	1.132	58.748	0.076	7.555	
Omitting Deng et al	1.371	0.426	0.166	0.986	50.697	0.076	7.606	
Omitting Gao et al	1.514	0.266	0.070	0.988	55.835	0.028	2.835	
Omitting Gil-Rodrigo et al	-0.939	-0.334	0.127	1.228	55.272	0.096	9.646	
Omitting Li, Li, Li et al	0.364	0.095	0.009	1.134	57.529	0.082	8.165	
Omitting Liu, Liu, Song et al	0.164	0.019	0.000	1.043	59.526	0.023	2.333	
Omitting McCullough et al	-1.327	-0.427	0.169	1.048	43.823	0.100	9.983	
Omitting Rath et al	-1.428	-0.410	0.167	1.077	55.500	0.077	7.689	
Omitting Shi et al	1.848	0.774	0.403	0.785	37.082	0.091	9.053	
Omitting Szekely et al	-0.561	-0.152	0.024	1.099	59.505	0.058	5.759	
Omitting He et al	0.350	0.086	0.008	1.111	58.356	0.071	7.115	
Omitting Zhang et al	0.886	0.203	0.041	1.045	57.330	0.049	4.914	
Omitting Zhou et al	-0.807	-0.265	0.075	1.164	58.602	0.084	8.369	
Omitting Zou et al	-0.218	-0.103	0.012	1.224	59.550	0.090	8.978	

Note how Shi et al and McCullough et al are outliers. (Baujat Plot)

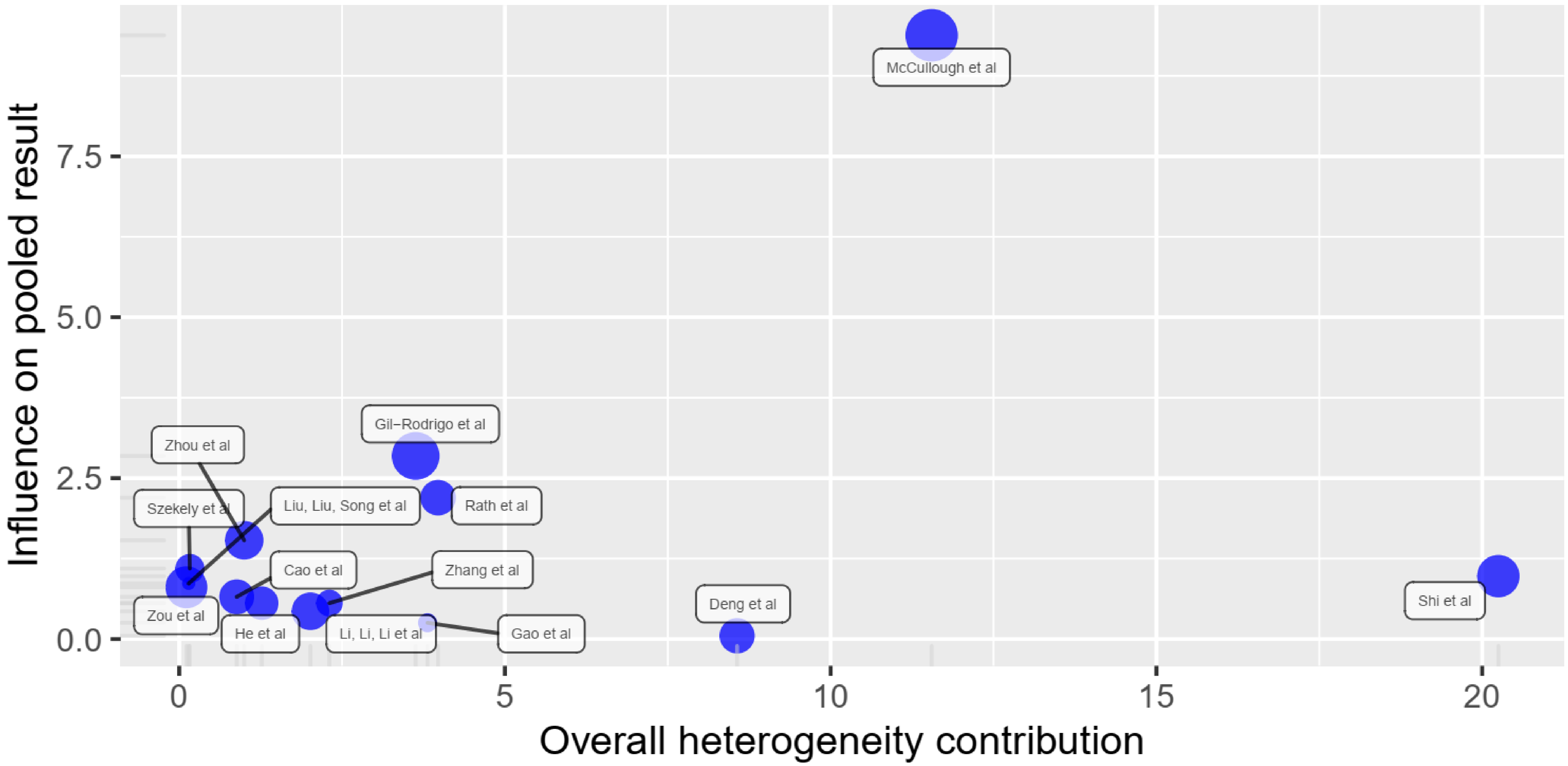

Baujat Diagnostics (sorted by Heterogeneity Contribution)

	HetContrib	InfluenceEffectSize
Omitting Shi et al	20.248	0.978
Omitting McCullough et al	11.551	9.380
Omitting Deng et al	8.564	0.052
Omitting Rath et al	3.974	2.197
Omitting Gao et al	3.812	0.253
Omitting Gil-Rodrigo et al	3.630	2.845
Omitting Zhang et al	2.306	0.559
Omitting Li, Li, Li et al	2.015	0.432
Omitting He et al	1.268	0.557
Omitting Zhou et al	0.998	1.535
Omitting Cao et al	0.884	0.656
Omitting Szekely et al	0.164	1.096
Omitting Liu, Liu, Song et al	0.146	0.864
Omitting Zou et al	0.111	0.805
